# Engineered assembly of water-dispersible nanocatalysts enables low-cost and green CO_2_ capture

**DOI:** 10.1038/s41467-022-28869-6

**Published:** 2022-03-10

**Authors:** Masood S. Alivand, Omid Mazaheri, Yue Wu, Ali Zavabeti, Andrew J. Christofferson, Nastaran Meftahi, Salvy P. Russo, Geoffrey W. Stevens, Colin A. Scholes, Kathryn A. Mumford

**Affiliations:** 1 Department of Chemical Engineering, The University of Melbourne, Melbourne, Vic 3010 Australia; 2 School of Agriculture and Food, Faculty of Veterinary and Agricultural Sciences, The University of Melbourne, Melbourne, Vic 3010 Australia; 3 School of Science, RMIT University, Melbourne, Vic 3001 Australia; 4 ARC Centre of Excellence in Exciton Science, School of Science, RMIT University, Melbourne, Vic 3000 Australia

**Keywords:** Carbon capture and storage, Chemical engineering, Metal-organic frameworks

## Abstract

Catalytic solvent regeneration has attracted broad interest owing to its potential to reduce energy consumption in CO_2_ separation, enabling industry to achieve emission reduction targets of the Paris Climate Accord. Despite recent advances, the development of engineered acidic nanocatalysts with unique characteristics remains a challenge. Herein, we establish a strategy to tailor the physicochemical properties of metal-organic frameworks (MOFs) for the synthesis of water-dispersible core-shell nanocatalysts with ease of use. We demonstrate that functionalized nanoclusters (Fe_3_O_4_-COOH) effectively induce missing-linker deficiencies and fabricate mesoporosity during the self-assembly of MOFs. Superacid sites are created by introducing chelating sulfates on the uncoordinated metal clusters, providing high proton donation capability. The obtained nanomaterials drastically reduce the energy consumption of CO_2_ capture by 44.7% using only 0.1 wt.% nanocatalyst, which is a ∽10-fold improvement in efficiency compared to heterogeneous catalysts. This research represents a new avenue for the next generation of advanced nanomaterials in catalytic solvent regeneration.

## Abstract (web summary)

Catalytic solvent regeneration is of interest to reduce energy consumption in CO2 separation, however, the development of engineered nanocatalysts remains a challenge. Here, a new avenue is presented for the next generation of advanced metal-organic frameworks (MOFs) in energy-efficient CO2 capture.

## Introduction

The growing trend of fossil fuel consumption and anthropogenic CO_2_ emission has gained global attention as an urgent environmental issue^1,2^. To overcome this challenge, the Paris Agreement was signed in 2015 by consensus to keep the Earth’s temperature rise well below 2 °C by mid-century^3,4^. Despite this, CO_2_ emission has been relentlessly increasing and approaching 40 GtCO_2_/year due to persistent release from key industrial sectors^5^. A strategy to reduce CO_2_ emissions while keeping existing industrial assets is carbon capture and storage (CCS). However, given the limited number of large-scale CO_2_ capture plants and the noticeable reduction in fossil fuel prices, hundreds of CCS facilities will need to be constructed by 2030 for the successful achievement of the Paris Agreement targets^6^. Chemical solvent CO_2_ absorption–desorption, as the most viable and widely accepted technique for carbon separation, is not inherently green and its high energy demand for solvent regeneration indirectly contributes to global CO_2_ emissions. Additionally, the energy-intensive nature of separation strongly impacts the economics of the process, leading to a reluctance to invest in CCS projects. Hence, it is of high importance to deploy a diverse portfolio of energy-efficient and green CO_2_ capture technologies with net-zero emissions.

The remarkable energy consumption of CO_2_ separation is mainly attributed to the high solvent regeneration temperature (above 100 °C) required to accelerate CO_2_ desorption kinetics^7,8^. Recently, catalytic solvent regeneration has emerged as an interesting approach to effectively promote the CO_2_ desorption rate and reduce the required regeneration energy^9,10^. One of the distinguishing features of catalytic regeneration is its potential to critically reduce the CO_2_ desorption temperature below 100 °C and accordingly pave the way for utilizing lower grade heat resources, such as solar hot water, as a green approach for solvent regeneration^7,11^. In this regard, a wide range of commercial and synthetic heterogeneous catalysts have been tested for catalyst-aided solvent regeneration, though their low efficiency and operational difficulties are still a significant barrier toward large-scale implementation^11^.

Metal–organic frameworks (MOFs) are an emerging class of porous materials with highly favorable catalytic properties owing to their large pore volume, abundant metal sites, and tailorable structure^12,13^. These features have made MOFs potential candidates for developing advanced nanocatalysts. Yaghi et al. recently reported the synthesis of an acidic MOF-SO_4_ catalyst by treating a zirconium-based MOF (MOF-808) with aqueous sulfuric acid^14,15^. They showed the strong Brønsted acidity of MOF-808-SO_4_ originates from the chelating sulfates attached to the unsaturated metal clusters which release protons into the reaction medium. However, the microporosity of MOFs is still a major concern for the fabrication of super-efficient nanomaterials, particularly in catalysis^16–18^. Additionally, there are other acid-stable MOFs desired for the fabrication of MOF-SO_4_ nanocatalysts which have not yet been explored. To this end, it is of great interest to exploit advanced MOF-SO_4_ materials with targeted physicochemical features for low-temperature CO_2_ desorption, thereby taking a step further toward establishing green CO_2_ capture technologies and achieving the objectives of the Paris Climate Accord.

**Fig. 1:**
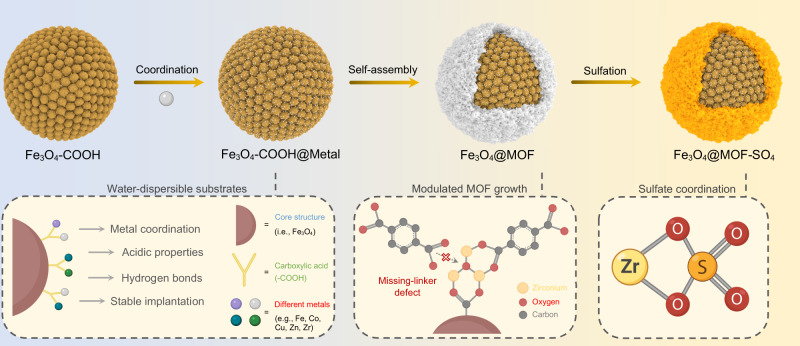
Synthetic strategy for engineering water-dispersible nanocatalysts. Schematic illustration of sulfated core–shell nanomaterials through modulated self-assembly of MOFs on the carboxylate-rich surface of Fe_3_O_4_–COOH nanoclusters. The acidic cover of magnetic nanocluster enables the creation of missing-ligand defects in the assembled structure and forms a hierarchical micro-mesoporous network. The coordination of chelating sulfate on metal clusters results in the formation of water-dispersible nanocatalysts with superacidity.

Herein, we present a new strategy for the fabrication of engineered water-dispersible nanocatalysts with hierarchical micro-mesoporous structures (Fig. 1 and Supplementary Fig. 4). In this technique, we used acidic Fe_3_O_4_ nanoclusters as a versatile substrate for the modulated self-assembly of different MOFs with broad structural diversity. The presence of carboxylates surrounding the core surface leverages the creation of active mesoporosity throughout the network of assembled MOFs. Specifically, the grown mesoporous shell facilitates the subsequent coordination of chelating sulfate moieties onto the metal clusters, thus allowing for a higher density of Brønsted acid sites. The accommodation of approachable acidic sites through the tailored hierarchical structure, as well as the nanofluidic aspect readily enables water-dispersible Fe_3_O_4_@MOF-SO_4_ nanocatalyst to actively participate in CO_2_ desorption reactions and drastically reduce energy consumption.

## Results

### Water-dispersible acidic Fe_3_O_4_ substrate

We first examined the potential of acidic Fe_3_O_4_ nanoclusters as favorable water-dispersible supports for the formation of advanced nanocatalysts. Ferric ammonium citrate, containing both iron (Fe^3+^) and citric acid, was selected as a cheap and environmentally benign precursor for the fabrication of carboxylated Fe_3_O_4_ nanoclusters (Fe_3_O_4_–COOH). One of the benefits of the citrate assembly approach is that the carboxylate groups on the surface of the nanocluster can serve as proton donor sites. Additionally, carboxylates can also provide stable hydrogen bonds to water molecules, allowing great dispersibility and facilitating the nanofluidic behavior of Fe_3_O_4_–COOH in aqueous solvents (Supplementary Fig. 5). The combination of these two features enables the Fe_3_O_4_–COOH to be simply utilized as a water-dispersible acidic catalyst during the continuous CO_2_ absorption–desorption operation without any modification to the process configuration.

**Fig. 2:**
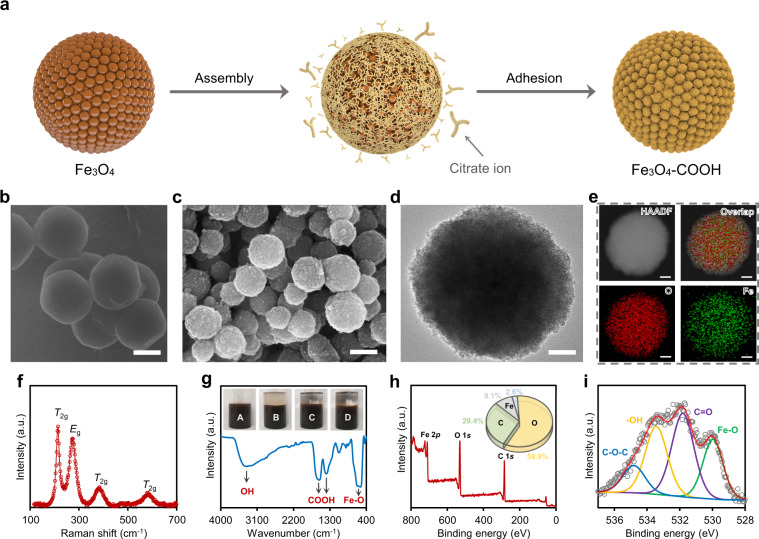
Synthesis of the water-dispersible magnetic substrate with acidic characteristics. **a** Schematic of the formation of nanoclusters in a one-pot synthesis methodology. **b** HIM and **c** SEM images of the acidic Fe_3_O_4_–COOH substrate. Scale bars are 200 nm. **d** TEM image of a single Fe_3_O_4_–COOH nanocluster and its corresponding **e** HAADF and EDX mapping. Scale bars are 50 nm. **f** Raman and **g** FTIR spectra of Fe_3_O_4_–COOH structure. Insets in **g** are the images of Fe_3_O_4_–COOH dispersion in pure water (**a**), followed by adding silicon oil (**b**), toluene (**c**), and diethyl ether (**d**). Before taking the picture, the mixtures were completely shaken and incubated for 15 min and nanocatalyst concentration was kept constant at 0.1 wt%; the transparency of the nonaqueous phase indicates the hydrophilicity of Fe_3_O_4_–COOH nanoclusters and their accumulation in the aqueous phase. **h** Full survey and **i** high-resolution XPS spectra showing the chemical moieties on the exterior surface of Fe_3_O_4_–COOH nanoclusters.

The surface modification is achieved via two steps by the assembly and adhesion of citrate ions to the Fe_3_O_4_ surface (Fig. 2a). Upon partial reduction of Fe^3+^ to Fe^2+^ in the non-aqueous solution and formation of single-crystal Fe_3_O_4_ nanoparticles, citrate ions easily attach to the surface owing to the strong coordination affinity between carboxylate groups and Fe^3+^/Fe^2+^ ions, resulting in aggregation of single nanoparticles to form large Fe_3_O_4_ nanoclusters^19,20^. Helium ion microscopy and scanning electron microscopy (SEM) showed the spherical clusters ranging in size from ⁓100 to 300 nm (Fig. 2b, c and Supplementary Fig. 6). Specifically, transmission electron microscopy (TEM) indicated the aggregated nanoclusters are composed of small adhesive Fe_3_O_4_ nanoparticles with about ⁓2-5 nm size (Fig. 2d). High-angle annular dark-field (HAADF), energy-dispersive X-ray spectroscopy (EDX) mapping, and elemental line scanning confirmed the homogeneous morphology of Fe_3_O_4_ nanoclusters with uniform Fe and O distribution throughout the structure (Fig. 2e and Supplementary Fig. 7). In addition, *T*_2g_ and *E*_g_ peaks were identified as the prominent vibrational modes in the Raman spectrum which confirm the successful formation of the Fe_3_O_4_ structure (Fig. 2f).

Different characterization methods were utilized to confirm the successful assembly of carboxylic acid groups (COOH) on the surface of Fe_3_O_4_–COOH nanoclusters. Fourier transform infrared spectroscopy (FTIR) displayed two characteristic peaks at ⁓1340 and ⁓1610 cm^−1^ corresponding to the carbonyl group (C=O) and C–OH stretching of the carboxylic acid group (Fig. 2g)^21^. X-ray photoelectron spectroscopy (XPS) revealed that the majority of Fe_3_O_4_–COOH surface is covered by O (⁓58.9%) and C (⁓29.4%), while the share of Fe was ⁓9.1% (Fig. 2h, i and Supplementary Fig. 8). In contrast, the elemental analysis showed the bulk is mainly composed of Fe (⁓87.0%), which is ⁓28-fold greater than that of C (⁓2.9%) in the bulk sample (Supplementary Fig. 9), suggesting that COOH groups have mainly accumulated on the external surface of the Fe_3_O_4_–COOH nanocluster.

The anchored citrate groups can subsequently favor the hydrophilic nature of Fe_3_O_4_–COOH, its electrostatic stabilization, and dispersibility in the aqueous media, due to the intense negative charge density of the surface. To further demonstrate the hydrophilicity of Fe_3_O_4_–COOH, its aqueous solution was mixed with silicon oil, toluene, and dichloromethane, and no Fe_3_O_4_–COOH was observed in the non-aqueous solvents, highlighting the catalytic potential of Fe_3_O_4_–COOH in phase induced areas, such as CO_2_ capture using liquid–liquid phase change solvents (Fig. 2g)^22^. These results indicate that Fe_3_O_4_–COOH can be viably used as a water-dispersible substrate for further surface modification, having tunable physicochemical motifs that are of interest for catalytic solvent regeneration in CO_2_ capture.

### Tailoring the properties of Fe_3_O_4_@MOF

**Fig. 3:**
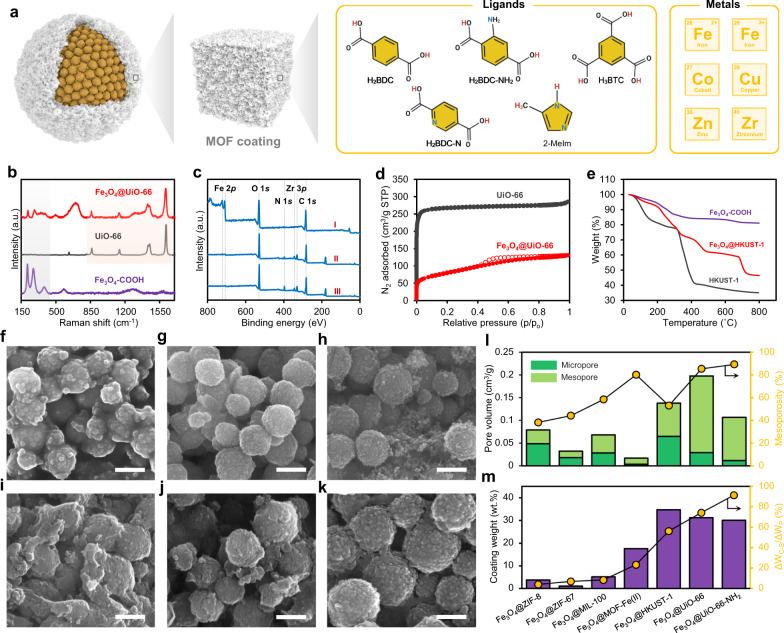
Structural tailoribility of MOFs via modulated self-assembly. **a** Schematic diagram of different metal ions and organic ligands used to grow various MOF coatings with unique physicochemical characteristics. **b** Raman spectra of the Fe_3_O_4_–COOH, UiO-66, and Fe_3_O_4_@UiO-66. **c** Full survey XPS spectra of Fe_3_O_4_@HKUST-1 (I), Fe_3_O_4_@UiO-66 (II), and Fe_3_O_4_@UiO-66-NH_2_ (III). **d** Nitrogen adsorption-desorption isotherms of UiO-66 and Fe_3_O_4_@UiO-66 at 77 K. **e** TGA profiles of the Fe_3_O_4_-COOH, HKUST-1, and Fe_3_O_4_@HKUST-1 under nitrogen atmosphere. SEM images of **f** Fe_3_O_4_@ZIF-8, **g** Fe_3_O_4_@ZIF-67, **h** Fe_3_O_4_@MOF-Fe(II), **i** Fe_3_O_4_@MIL-100(Fe), **j** Fe_3_O_4_@HKUST-1, and **k** Fe_3_O_4_@UiO-66. Scale bars are 200 nm. **l** Pore volume of the hierarchical Fe_3_O_4_@MOF materials with missing-linker defects and mesopore-induced network. **m** The coating weight and comparative weight reduction of various Fe_3_O_4_@MOF core–shells. The comparative weight reduction was calculated by the weight loss of core–shell material (ΔW_C-S_) divided by that of its corresponding pristine MOF (ΔW_P_) in the 100–350 °C range.

To appraise the modulated self-assembly of different MOFs on acidic Fe_3_O_4_–COOH substrate, seven MOFs, including ZIF-8, ZIF-67, MIL-100(Fe), MOF-Fe(II), HKUST-1, UiO-66, and UiO-66-NH_2_ with various metal (zinc (Zn), zirconium (Zr), cobalt (Co), copper (Cu), ferrous (Fe^2+^), and ferric (Fe^3+^)) and ligand (benzene-1,4-dicarboxylic acid (H_2_BDC), 2-aminoterephthalic acid (H_2_BDC-NH_2_), 2,5-pyridinedicarboxylic acid (H_2_BDC-N), benzene-1,3,5-tricarboxylic acid (H_3_BTC) and 2-methylimidazole (2-Melm)) combinations were utilized (Fig. 3a). After growing MOFs, the zeta potential of Fe_3_O_4_–COOH substrate in all cases changed from −43.1 mV to greater values (ranging from −1.7 to −19.5 mV), indicating that the surface of the substrate is primarily covered by metal–ligand assembly (Supplementary Fig. 10). This variation was due to the coordination of citrate ions and thereby diminution in the negative surface charge onto the nanocluster. In detail, the citrate groups of Fe_3_O_4_–COOH nanocluster can be easily dissociated and create a negatively charged surface, while hydroxyl groups of MOFs have a lower dissociation constant than those of carboxylates resulting in a decrease in the negative surface charge of Fe_3_O_4_@MOFs. The successful preparation of Fe_3_O_4_@MOF core–shell structure was further confirmed by TEM images, nitrogen adsorption–desorption analysis, FTIR, XRD, Raman spectroscopy, and XPS characterization (Fig. 3b–d and Supplementary Figs. 11–16).

SEM images were also employed to observe the exterior of Fe_3_O_4_@MOF materials. As shown in Fig. 3f–k, Fe_3_O_4_@MOFs exhibited almost different surface morphologies from highly uniformed spherical shapes, similar to that of pristine Fe_3_O_4_–COOH without MOF coating, to semispherical conjunct core-shell structure, like Fe_3_O_4_@MIL-100(Fe). This structural diversity is mainly imputed to the unique characteristic features of MOFs. As MOFs are created from different metal–ligand coordination, a series of frameworks with various physicochemical properties, crystal size, and surface roughness can be obtained (Supplementary Figs. 17–19). Furthermore, the high density of carboxylic acid sites on the rough surface of the substrate, as a potential nucleation site, can effectively manipulate the size and shape of the crystal, justifying the super small MOF crystals spotted on the exterior surface of core–shell structures compared to their pristine MOFs with large crystals.

In addition to the surface morphology, we investigated the porosity of Fe_3_O_4_@MOFs, particularly relative to those of pristine MOFs. As observed in Fig. 3l, the total pore volume of core-shell materials increased to ⁓0.02–0.20 cm^3^/g, whereas a negligible porosity was detected for the acidic core itself. The difference in the porosity of Fe_3_O_4_@MOFs can likely be attributed to the different pore architectures of MOFs on the exterior shell side. Notably, a comparison of pore volume exhibited that Fe_3_O_4_@UiO-66 has the maximum pore volume of ⁓0.20 cm^3^/g among core–shell materials, highlighting the determining role of MOF type in the created porosity. Interestingly, we found that the modulated core–shell nanomaterials benefit from a hierarchical micro-mesoporous structure, while pristine MOFs are generally microporous materials (Supplementary Figs. 20–22). For example, the mesoporosity, i.e., mesopore divided by total pore volume, of the modulated UiO-66 shell (⁓85.4%) was much greater than that of pristine UiO-66 (⁓11.1%) synthesized by the conventional procedure. The created mesoporosity in the coating could be ascribed to the induced missing-linker defects during the modulated self-assembly of MOFs on the acidic core. Similar to the carboxylic acid linkers used for the synthesis of MOFs, Fe_3_O_4_–COOH can partially play the role of ligand, change the bonding energy of metal–ligand coordination and manipulate the MOF formation mechanism. Accordingly, the MOF layers cannot smoothly grow on the carboxylic acid-rich substrate which can create mesoporosity and engineer the pore structure by hindering the bridging linkers and changing the coordination environment during their modulated self-assembly.

To confirm the role of missing-linker defects in the formation of engineered core–shell materials, thermogravimetric analysis was used (Fig. 3e and Supplementary Fig. 23). Because of the thermal decomposition of organic ligands from 100 to 350 °C, the weight reduction ratio of core–shell structure (ΔW_C-S_) to pristine MOF (ΔW_P_) in this temperature range was considered as an indicator of missing-linker deficiency^23^. Although Zn-, Co-, and Fe-based MOFs showed a high linker deficiency, the predominance of metal coordination through the network could have a negative impact on the self-assembly process, resulting in the poor formation of MOF structure (Fig. 3m). This is compatible with their low coating layer weights. In contrast, both Cu- and Zr-based MOFs displayed a good linker deficiency (⁓56.2–91.5%), as well as high coating weight (⁓30.1–34.7 wt%) and homogenous coating (Supplementary Figs. 24–27), likely due to the optimum surface energy of Fe_3_O_4_–COOH@Cu and Fe_3_O_4_–COOH@Zr compared to their corresponding organic ligands^24–26^. These results demonstrate that the self-assembly of MOFs on Fe_3_O_4_–COOH can be used as a simple platform to prepare advanced mesopore-induced core–shell materials with tailored properties, specifically more defects, and unsaturated metal sites.

### The formation of Fe_3_O_4_@MOF-SO_4_

**Fig. 4:**
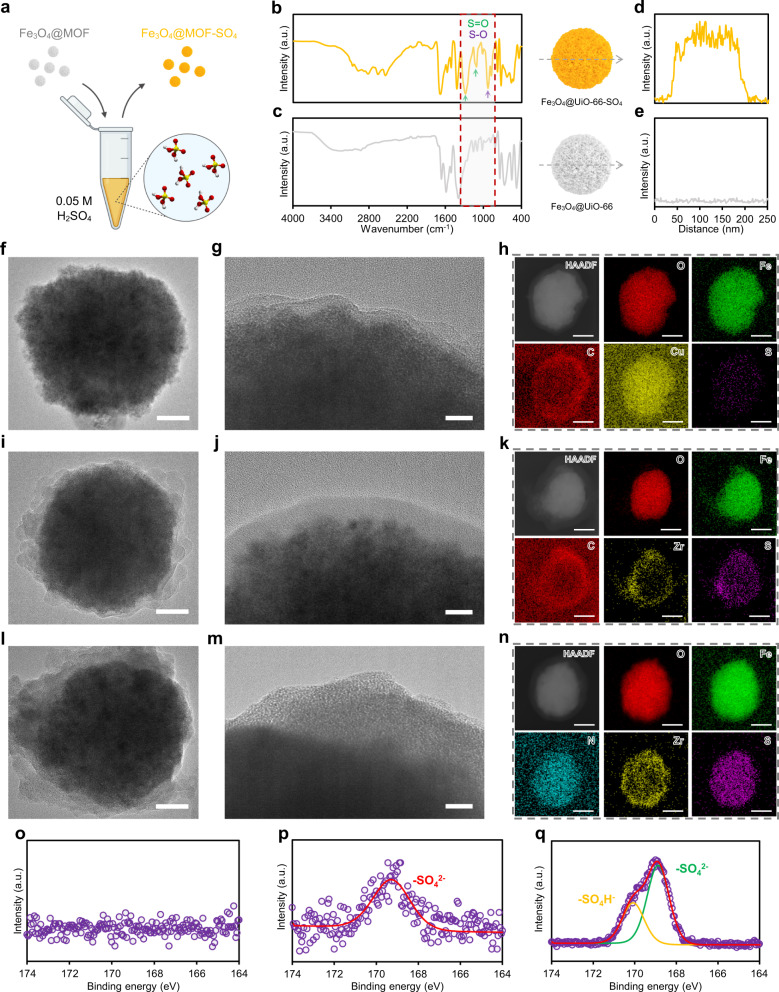
Coordination of chelating sulfate species in defected core–shell structure. **a** Schematic illustration of the preparation of water-dispersible Fe_3_O_4_@MOF–SO_4_ nanocatalyst via aqueous sulfation process. **b**, **c** FTIR spectra, and **d**, **e** sulfur line scanning profile of Fe_3_O_4_@UiO-66 and Fe_3_O_4_@UiO-66-SO_4_. TEM, HAADF, and EDX images of **f**–**h** Fe_3_O_4_@HKUST-SO_4_, **i**–**k** Fe_3_O_4_@UiO-66-SO_4_, and **l**–**n** Fe_3_O_4_@UiO-66-NH_2_–SO_4_. Scale bars are 50 (**f**, **i**, **l**), 10 (**g**, **j**, **m**), and 100 nm (**h**, **k**, **n**). High-resolution XPS spectra of sulfur species in **o** Fe_3_O_4_@HKUST-SO_4_, **p** Fe_3_O_4_@UiO-66-SO_4_, and **q** Fe_3_O_4_@UiO-66-NH_2_-SO_4_.

Defect-engineered MOFs are promising platforms for developing advanced functional nanomaterials for various catalytic applications, including photo- and electrocatalysis, as they can provide active metal sites with a strong affinity towards a broad range of functional moieties^12^. We, therefore, with respect to our interest in acidic nanocatalysts, treated the engineered Fe_3_O_4_@MOFs with diluted sulfuric acid to introduce sulfate moieties through their defected structures. The general procedure for Fe_3_O_4_@MOF sulfation in the present study is schematically demonstrated in Fig. 4a and Supplementary Fig. 28. The primary advantage of the reported sulfation method is that Fe_3_O_4_@MOF-SO_4_ can be simply prepared by dispersing Fe_3_O_4_@MOFs into the aqueous solution of sulfuric acid (0.05 M, pH ⁓1.3) at room conditions which makes it a viable approach for large-scale implementation. FTIR analysis of Fe_3_O_4_@MOF-SO_4_ disclosed the presence of sulfur compounds in 800–1300 cm^−1^ region, including both S–O (⁓800–950 cm^−1^) and S=O (⁓1000–1300 cm^−1^) bonds, thus confirming that SO_4_^−2^ species were successfully coordinated with active metal sites (Fig. 4b, c and Supplementary Fig. 29)^27^. In addition, elemental line scanning profiles also revealed that there is a good distribution of sulfur across the treated core–shells, while no sulfur was detected before acid treatment (Fig. 4d, e). Since the sulfates can take different coordination positions on the surface of uncoordinated metal clusters, missing-linker deficiency in defect-engineered core–shells can positively manipulate the chelating mode of sulfate by providing additional space, resulting in the improved sulfation yield.

To explore the versatility of the aqueous sulfation method for Fe_3_O_4_@MOF, Fe_3_O_4_@HKUST-1, Fe_3_O_4_@UiO-66, and Fe_3_O_4_@UiO-66-NH_2_ with the highest pore volumes were selected for post-treatment. TEM analysis illustrated the Fe_3_O_4_@MOF structures well preserved their core–shell structures at low pH values (i.e., ⁓1.3), with a shell thickness changing from ⁓10 nm in Fe_3_O_4_@HKUST-SO_4_ (Fig. 4f, g) to ⁓20 nm in Fe_3_O_4_@UiO-66–SO_4_ (Fig. 4i, j) and ⁓40 nm in Fe_3_O_4_@UiO-66-NH_2_–SO_4_ (Fig. 4l, m). HAADF and EDX maps reconfirmed the homogeneous distribution of metallic and organic elements corresponding to their MOF structures (Fig. 4h, k, n). Nevertheless, it was found that the amount of sulfur elements varies among Fe_3_O_4_@MOF–SO_4_ materials, and Fe_3_O_4_@HKUST–SO_4_ possesses the least sulfur content when compared with those of Zr-based core–shell structures. This observation was further examined by high-resolution XPS analysis, showing the sulfur species on the exterior surface of Fe_3_O_4_@MOF–SO_4_ materials (Fig. 4o–q and Supplementary Fig. 30). Notably, XPS peaks did not appear at 164–174 eV (the typical range of binding energy for sulfur, S 2*p*), validating the low sulfur signals in its EDX. These results suggest that the chemical stability of Fe_3_O_4_@MOF materials is a key contributor to the aqueous sulfation process. For instance, HKUST-1 framework is composed of Cu^2+^ ion pairs chelated by carboxylate bridges with paddle-wheel units; however, the high concentration of protons at low pH values can potentially accelerate the hydrolysis of Cu–O bonds, resulting in the partial disassembly of MOF crystals^28^. Similarly, Fe_3_O_4_@HKUST-1 could not entirely preserve its structural stability at harsh acidic conditions, as the shell side partially dissociated (Supplementary Fig. 31) and the average shell thickness remarkably diminished after the sulfation process. Furthermore, the unstable Cu bond could prohibit both bridging and chelating mode of sulfate coordination which verifies the poor efficiency of Fe_3_O_4_@HKUST-1 sulfation. In contrast, both Fe_3_O_4_@UiO-66–SO_4_ and Fe_3_O_4_@UiO-66-NH_2_–SO_4_ exhibited that sulfate species were successfully coordinated to the Zr metals, owing to the tolerance of Zr–O bond in a broad pH range from 1 to 10^29^. It is worth noting that good stability was also observed for Fe_3_O_4_–COOH nanoclusters at pH 1–3, whereas conventional Fe_3_O_4_ nanoparticles immediately digested into the acidic solution at the same conditions. This difference may be due to the citrate groups covering the surface of nanoparticles with lower p*K*a values (⁓3.0–5.5) than those of unfunctionalized Fe_3_O_4_ nanoparticles (⁓5.3–8.8), which could be deprotonated at low pH values and prevent the subsequent disassembly of Fe–O structure.

### Catalytic CO_2_ desorption performance

**Fig. 5:**
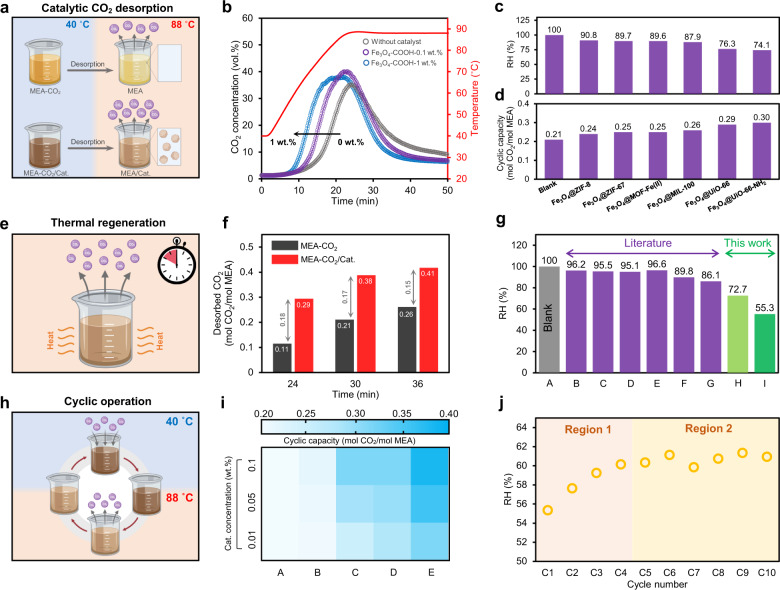
Application of water-dispersible nanocatalysts for energy-efficient CO_2_ capture. **a** Schematic of amine solvent regeneration with and without using a catalyst (Cat.). **b** CO_2_ desorption profile of amine solution with different concentrations of Fe_3_O_4_–COOH. **c** Relative heat duty (RH) and **d** cyclic CO_2_ uptake capacity of amine solution in the presence of various water-dispersible core–shell nanomaterials. **e** Schematic of catalytic CO_2_ desorption during thermal solvent regeneration. **f** The effect of time on the accumulative desorbed CO_2_ with and without using Fe_3_O_4_@UiO-66–SO_4_. **g** RH of amine solution without (**a**) and with heterogeneous catalysts (Al_2_O_3_ (**b**), V_2_O_5_ (**c**), H-Beta (**d**), HZSM-5 (**e**), SO_4_^2^^−^/ZrO_2_/Al_2_O_3_ (**f**), SO_4_^2−^/ZrO_2_/SBA-15 (**g**)), water-dispersible Fe_3_O_4_–COOH (**h**), and Fe_3_O_4_@UiO-66-SO_4_ (**I**). **h** Schematic of cyclic CO_2_ absorption–desorption of catalyst-aided solvent regeneration. **i** The effect of catalyst concentration on the cyclic CO_2_ capacity; blank (**a**), with H-Bata (**b**), Fe_3_O_4_–COOH (**c**), Fe_3_O_4_@UiO-66 (**d**), and Fe_3_O_4_@UiO-66-SO_4_ (**e**). **j** Recyclability of the Fe_3_O_4_@UiO-66–SO_4_ during consecutive CO_2_ absorption–desorption cycles. The amine solution in all cases is 5 M MEA in water and no catalyst was used for the blank solvent. The concentration of catalyst was fixed at 0.1 wt% in (**c**, **d**, **f**, **g**, **j**).

Catalytic solvent regeneration, a recently emerged technique, has garnered wide attention, because of its low-temperature operation and high energy efficiency. The acidic nanocatalysts can act as a good proton donor, supplying the excess amount of protons required for the carbamate breakdown reaction, and promote CO_2_ desorption at temperatures less than 100 °C (the boiling temperature of water at atmospheric pressure)^30^. To illustrate that the prepared water-dispersible nanomaterials can be used to accelerate CO_2_ desorption reactions, we first examined the catalytic performance of acidic Fe_3_O_4_–COOH nanoclusters during the regeneration of CO_2_-rich monoethanolamine (MEA, 5 M) at 88 °C (Fig. 5a). As shown in Fig. 5b, adding a small amount of Fe_3_O_4_–COOH (0.1 wt%) significantly increased the kinetics of CO_2_ desorption, resulting in ⁓27.3% less energy consumption, when compared with that of blank MEA solution without using any catalyst. By increasing the amount of Fe_3_O_4_–COOH from 0.1 to 1 wt%, CO_2_ was released more quickly from the solvent; however, the energy efficiency parameter (i.e., the absolute of relative heat duty reduction versus the amount of catalyst used) significantly dropped from 2.73 to 0.66, respectively. Thus, a concentration of 0.1 wt% was established to be the optimal value of water-dispersible nanocatalysts, almost 10 times less than previously reported values, owing to the abundant active acidic sites on the surface of Fe_3_O_4_–COOH, as well as the Brownian motion and nanofluidic behavior of magnetic nanoclusters in the solvent^31,32^. These results highlight the potential of Fe_3_O_4_–COOH as a versatile substrate for the synthesis of acidic water-dispersible nanocatalysts which can be easily added at low concentrations during the continuous operation of CO_2_ absorption–desorption processes.

Besides the CO_2_ desorption performance of Fe_3_O_4_–COOH, the catalytic behavior of defect-engineered Fe_3_O_4_@MOFs was explored. Figure 5c, d shows that all prepared core–shell materials improved the kinetics of CO_2_ desorption, ranging from Fe_3_O_4_@ZIF-8 to Fe_3_O_4_@HKUST-1 with the least and the best performance, respectively. From these observations, it appears that a MOF coating had a negative impact on the catalytic efficiency of Fe_3_O_4_–COOH substrates. Indeed, Fe_3_O_4_@HKUST-1 (as the best of core–shell materials) depicted a relative heat duty of 74.1%, whereas 72.7% was recorded when solely using Fe_3_O_4_–COOH. This could be attributed to the replacement of Brønsted acid sites (i.e., carboxylates) with Lewis acid sites (uncoordinated metal clusters) with less proton donation capability^33^. Contrarily, enriching Brønsted acid sites through the hierarchical structure of core–shell structures (i.e., Fe_3_O_4_@MOF–SO_4_) resulted in a distinct catalytic performance. The Fe_3_O_4_@UiO-66–SO_4_ succeeded to desorb 80.9% more CO_2_ compared with that of the blank solution at similar operating conditions. In addition, we found a decreasing trend in the differential desorbed CO_2_ with the regeneration time (from 24 to 36 min), highlighting the substantial influence of catalyst on the kinetics of CO_2_ desorption (Fig. 5e, f). To further investigate the catalytic performance of the Fe_3_O_4_@UiO-66–SO_4_, its corresponding relative heat duty was compared with those of Fe_3_O_4_–COOH and commercialized solid acid catalysts, including metal oxides (Al_2_O_3_, V_2_O_5_) and zeolites (H-Beta and HZSM-5) (Fig. 5g and Supplementary Fig. 32). The Fe_3_O_4_@UiO-66–SO_4_ exhibited the lowest required energy for the regeneration of CO_2_-rich MEA solution with a relative heat duty of 55.3%, mainly due to its large pore volume, predominant mesoporous structure, abundant Brønsted acid sites, and nanofluidic behavior (Supplementary Figs. 33 and 34). These findings also suggested that the performance of other prevalent heterogeneous nanocatalysts is not comparable with the water-dispersible materials at low catalyst concentrations (less than 0.1 wt%).

To specifically explore the performance of water-dispersible nanomaterials at low concentrations (0.01, 0.05, and 0.1 wt%), their cyclic CO_2_ absorption–desorption capacity was measured (Fig. 5h, i). As discussed, H-Beta zeolite failed to display any sensible promotion throughout the course of the CO_2_ desorption operation. Unlike H-Beta, all Fe_3_O_4_–COOH, Fe_3_O_4_@UiO-66, and Fe_3_O_4_@UiO-66–SO_4_ demonstrated comparable cyclic CO_2_ capacity, even at an extremely low concentration of 0.01 wt%. For instance, adding Fe_3_O_4_@UiO-66–SO_4_ nanocatalysts increased the cyclic capacity of CO_2_ from 0.21 mol CO_2_/mol MEA in the blank solution to 0.30, 0.33, and 0.38 mol CO_2_/mol MEA with 0.01, 0.05, and 0.1 wt% concentrations of nanocatalyst, respectively, which are comparable with those of commercialized catalysts with ⁓10- to ⁓100-fold higher concentrations (⁓1.0–1.1 wt%)^34^. Since acidic catalyst allows for enhanced cumulative CO_2_ desorption during the solvent regeneration process, the solvent can absorb more CO_2_ in the next absorption cycle, leading to the better performance of the solvent (in terms of equilibrium and kinetics) in the absorption column. The stability of Fe_3_O_4_@UiO-66–SO_4_ was assessed via ten cycles of consecutive CO_2_ absorption–desorption operation (Fig. 5j). As seen, the relative heat duty of MEA solution increased by only ⁓9% over the first four cycles (region 1) and remained stable throughout the last six cycles (region 2). In addition, no significant changes were observed in the XPS spectra, XRD patterns, and SEM images of Fe_3_O_4_@UiO-66–SO_4_, before and after the cyclic operation (Supplementary Fig. 35), confirming the excellent recyclability of these water-dispersible nanocatalysts.

**Fig. 6:**
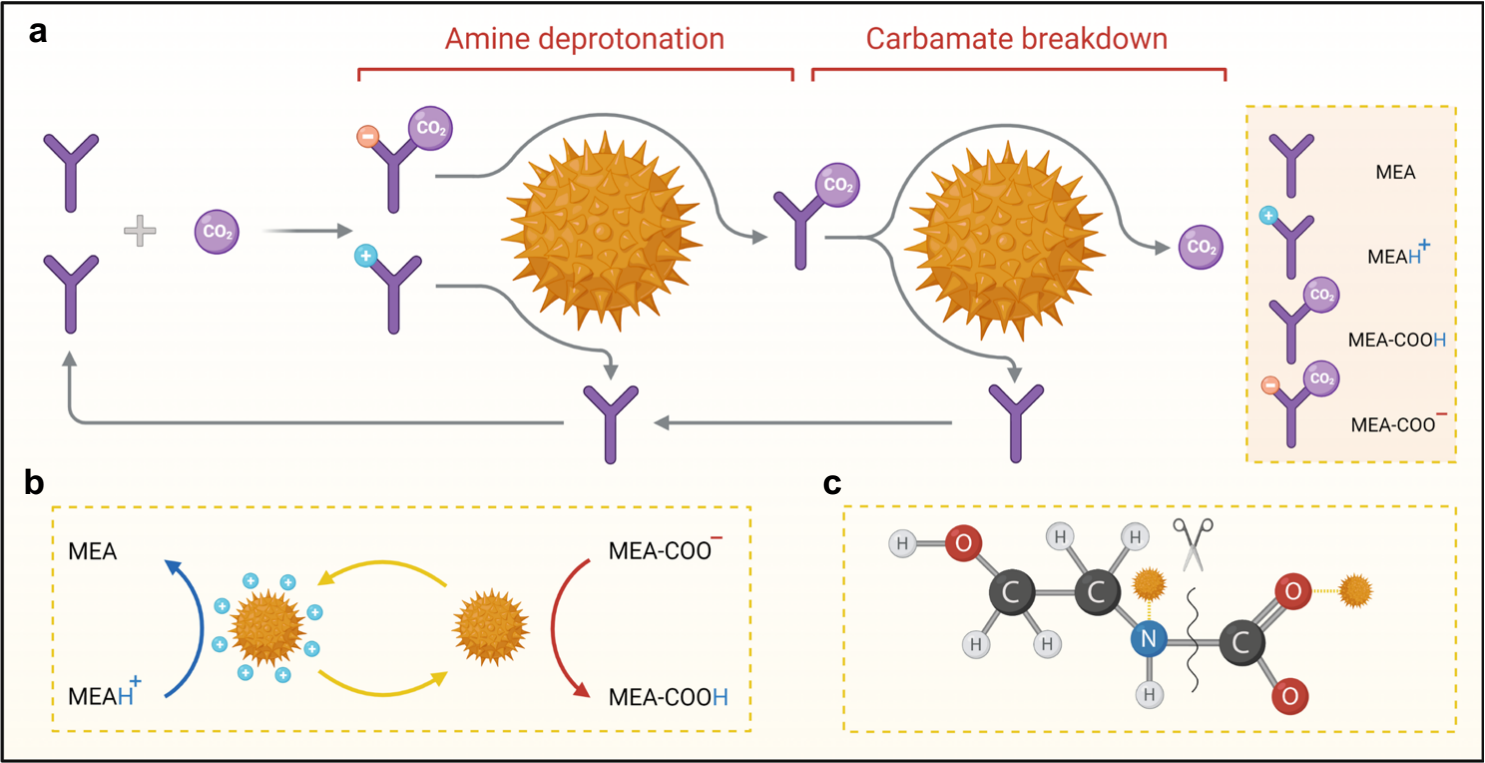
Catalytic CO_2_ desorption mechanism. **a** The acidic nanocatalyst enables an accelerated proton transfer from protonated amine (MEAH^+^) to carbamate (MEA-COO^−^), and facilitated the decomposition of the zwitterion (MEA-COOH). It resulted in enhanced CO_2_ desorption at low operating temperatures with the lower required energy. **b** The possible proton transfer routes from MEAH^+^ to MEA-COO^−^ using the Brønsted acid sites of water-dispersible nanocatalyst. **c** The proposed carbamate breakdown mechanism in the presence of acidic nanocatalyst resulted in reusable MEA and CO_2_.

Besides all targeted features of Fe_3_O_4_@UiO-66–SO_4_ for catalyst-aided solvent regeneration, its distinguished catalytic performance, particularly when compared with Fe_3_O_4_–COOH, could be assigned to its special proton donation mechanism. Throughout the solvent regeneration, CO_2_ molecules are generally released by the carbamate breakdown reaction. However, the yield of this reaction is highly dependent on the number of active protons in the reaction medium supplied by the amine deprotonation reaction (Fig. 6)^35^. Owing to the endothermic nature of all reactions, a high operating temperature (⁓120–140 °C) is required for spontaneous proton transfer and bond cleavage, resulting in high-quality steam use and subsequently high energy consumption^36^. In Fe_3_O_4_@UiO-66–SO_4_, the adsorbed water molecules on the surface of uncoordinated Zr clusters can participate in a hydrogen bond with a sulfate moiety chelated to another neighboring Zr center^14^. This specific arrangement of sulfate and water moieties results in the formation of superacid sites (H_0_ ≤ −14.5; see Supplementary Table 1) with distinct proton donation ability, accelerating the carbamate breakdown reaction and allowing for enhanced CO_2_ desorption at low regeneration temperatures (less than 100 °C). We note that all water-dispersible nanocatalysts could successfully recover their released protons during the CO_2_ absorption process, as compared to heterogeneous solid acid catalysts which need to be protonated via an acid washing processes. The results indicate that the unique privileges of water-dispersible nanomaterials (i.e., ease of use, low operating concentration, and high efficiency) can potentially make the implementation of catalytic solvent regeneration industrially affordable.

**Fig. 7:**
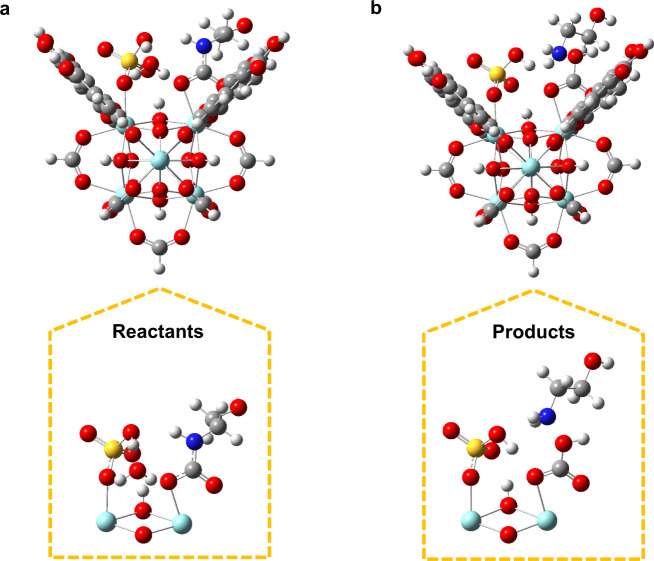
Reactants and products for the decomposition of carbamate on a Fe_3_O_4_@UiO-66–SO_4_ defect active site. **a** Reactants. **b** Products. The full fragment is shown in the upper panel, and a rotated view of the defect active site alone is shown in the lower panel. Zirconium, sulfur, oxygen, nitrogen, carbon, and hydrogen are colored cyan, yellow, red, blue, gray, and white, respectively.

In order to gain a greater understanding of the carbamate breakdown catalyzed by Fe_3_O_4_@UiO-66–SO_4_, density functional theory (DFT) calculations were performed. Starting from a defect active site with HSO_4_^−^ and H_2_O coordinating the two Zr atoms, the addition of carbamate resulted in the displacement of the H_2_O by the carbamate carboxylate group (Fig. 7a). This configuration places the HSO_4_^−^ hydroxyl oxygen within ~3 Å of the carbamate nitrogen. While the exact order of proton transfer is unclear, the protonation of the carbamate nitrogen by either the HSO_4_^−^ or the nearby H_2_O resulted in the breakage of the N–C bond, coupled with the formation of a C–O bond from the H_2_O, leading to stable products of MEA and HCO_3_^−^ (Fig. 7b). Attempts to protonate the carbamate nitrogen and break the C–N bond in the absence of the water molecule were unsuccessful, suggesting that the presence of H_2_O and the formation of HCO_3_^−^ as a product are essential in the catalytic reaction.

## Discussion

In summary, we have developed a new and effective methodology to synthesize a series of water-dispersible nanocatalysts with engineered properties and nanofluidic behavior for low-temperature CO_2_ desorption. A promising feature of the current method is that the carboxylate-rich surface of Fe_3_O_4_–COOH nanoclusters allows for the modulated growth of MOFs on its structure. Importantly, we observed a transition from the microporous structure in pristine MOFs to a hierarchical micro-mesoporous network in Fe_3_O_4_@MOFs. Different metal ion and organic ligand combinations can be selected for the tailored self-assembly of MOFs on the magnetic core, resulting in a broad diversity of physicochemical properties. The obtained materials with structural defects and active metal centers can serve as a potential platform for the coordination of chelating sulfates with superacidity. Enhanced kinetics of CO_2_ desorption was obtained for all Fe_3_O_4_–COOH, Fe_3_O_4_@MOF, and Fe_3_O_4_@MOF–SO_4_ materials. As a proof of concept, Fe_3_O_4_@UiO-66-SO_4_ exhibited the best catalytic performance (44.7% reduction in energy consumption) with excellent recyclability (⁓9% loss over five cycles) using a low catalyst concentration of only 0.1 wt%. The comprehensive characterizations revealed that the missing-linker defects and superacidic sites through the hierarchical network of Fe_3_O_4_@UiO-66–SO_4_ are key contributors to its advanced proton donation capability and thus accelerated CO_2_ desorption performance. We anticipate that this approach will open up a new avenue to the synthesis of water-dispersible nanocatalysts in the area of catalytic CO_2_ absorption–desorption and take one further step toward the utilization of green energy (e.g., solar hot water) in the portfolio of energy-efficient CO_2_ capture.

## Methods

### Synthesis of acidic Fe_3_O_4_–COOH nanoclusters

Typically, ferric ammonium citrate (3.25 g) and NaOAc (6 g) were dissolved in 100 mL EG under vigorous stirring at room temperature. The formed hazy solution was transferred to a stainless-steel autoclave (150 mL capacity) and heated at 200 °C for 10 h. Then, the autoclave was gradually cooled to room temperature and the black solid precipitants were separated via the application of an external magnet. The obtained nanoclusters were repeatedly washed with acetone, ethanol, and water to remove unreacted or physically attached molecules. During each washing step, the nanoclusters were suspended in the solvent, ultrasonicated for 30 min, and separated by an external magnet. Finally, the resultant materials were dried at 80 °C overnight under a severe vacuum and labeled as Fe_3_O_4_–COOH.

### Synthesis of conventional Fe_3_O_4_ nanoparticles

To compare the structural properties of Fe_3_O_4_–COOH nanoclusters with a benchmark, conventional Fe_3_O_4_ nanoparticles were synthesized according to the previously reported co-precipitation method with minor modifications^37^. Firstly, 298.5 mg of FeCl_2_·4H_2_O and 810 mg of FeCl_3_·6H_2_O were dissolved in 100 mL of water and sonicated for 15 min. The temperature was increased to 60 °C, and while keeping the mixture under vigorous agitation, NH_4_OH solution was added as an oxidation agent to trigger the iron oxide precipitation. When the pH of the solvent reached 11, the temperature was increased to 80 °C and held for 2 h. The black precipitates were separated by an external magnet and washed with a copious amount of water to remove extra NH_4_OH reactants and OH^−^ ions. Eventually, the obtained products were dried in a vacuum oven at 80 °C overnight, labeled as Fe_3_O_4_ nanoparticles and stored for future use.

### Synthesis of Fe_3_O_4_@ZIF-8

For the synthesis of Fe_3_O_4_@ZIF-8 core–shell particles with a magnetic core, Fe_3_O_4_–COOH nanoclusters were first dispersed in 100 mL methanol (5 mg/mL) under vigorous stirring for 30 min. Then, 0.325 g of Zn(NO_3_)_2_·6H_2_O was added and the suspension was sonicated for 1 h to facilitate zinc metal ion coordination on the carboxylate groups of the Fe_3_O_4_–COOH cluster surface. For the self-assembly of the ZIF-8 shell, 100 mL of 2-MeIm solution (2.82 mg/mL in methanol) was added dropwise to the suspension and the mixture was stirred at room temperature for 12 h. The fabricated Fe_3_O_4_@ZIF-8 core–shell particles were separated by an external magnet. For the washing process, Fe_3_O_4_@ZIF-8 particles were sonicated in methanol for 15 min, while the solvent was replaced three times. The particles produced were vacuum dried at 120 °C overnight and labeled as Fe_3_O_4_@ZIF-8.

### Synthesis of Fe_3_O_4_@ZIF-67

For the synthesis of Fe_3_O_4_@ZIF-67 core–shell particles with a magnetic core, Fe_3_O_4_–COOH nanoclusters were first dispersed in 100 mL methanol (50 mg/mL) under vigorous stirring for 30 min. Then, 0.5 g of Co(NO_3_)_2_·6H_2_O was added, and the suspension was sonicated for 1 h to facilitate cobalt metal ion coordination on the carboxylate groups of the Fe_3_O_4_–COOH cluster surface. For the self-assembly of the ZIF-67 shell, 100 mL of 2-MeIm solution (5.6 mg/mL in methanol) was added dropwise to the suspension and the mixture was stirred at room temperature for 24 h. The fabricated Fe_3_O_4_@ZIF-67 core–shell particles were separated by an external magnet. For the washing process, Fe_3_O_4_@ZIF-67 particles were sonicated in methanol for 15 min, and the solvent was replaced three times. The particles were vacuum dried at 120 °C overnight and labeled as Fe_3_O_4_@ZIF-67.

### Synthesis of Fe_3_O_4_@MIL-100(Fe)

For the synthesis of Fe_3_O_4_@MIL-100(Fe) core–shell particles with a magnetic core, Fe_3_O_4_-COOH nanoclusters (5 mg/mL) were first dispersed in 100 mL of ethanol under vigorous stirring for 30 min. Then, 0.464 g of FeCl_3_·6H_2_O was added and the suspension was sonicated for 1 h to facilitate iron metal ion coordination on the carboxylate groups of the Fe_3_O_4_-COOH cluster surface. The obtained suspension was transferred to a 250 mL round bottom flask and heated at 70 °C using an external oil bath with a magnetic stirrer. After 1 h, 100 mL of H_3_BTC solution (3.61 mg/mL in water) was added dropwise to the suspension and the mixture was stirred at 70 °C for a further 6 h to homogeneously grow the MIL-100(Fe) shell. The fabricated Fe_3_O_4_@MIL-100(Fe) core–shell particles were naturally cooled to room temperature and separated by an external magnet. For the washing process, Fe_3_O_4_@MIL-100(Fe) particles were sonicated three times in ethanol for 15 min. The particles were vacuum dried at 110 °C overnight and labeled as Fe_3_O_4_@MIL-100(Fe).

### Synthesis of Fe_3_O_4_@MOF–Fe(II)

For the synthesis of Fe_3_O_4_@MOF–Fe(II) core–shell particles with a magnetic core, Fe_3_O_4_–COOH nanoclusters were first dispersed in 50 mL water (10 mg/mL) under vigorous stirring for 30 min. Then, 0.278 g of FeSO_4_ was added and the suspension was sonicated for 1 h to facilitate iron metal ion coordination on the carboxylate groups of the Fe_3_O_4_–COOH cluster surface. The obtained suspension was transferred to a 250 mL round bottom flask and heated at 130 °C using an external oil bath with a magnetic stirrer. After 1 h, 150 mL of H_2_BDC-N solution (1.67 mg/mL in DMF) was added dropwise to the suspension and the mixture was stirred at 130 °C for a further 4 h to homogeneously grow the MOF-Fe(II) shell. The fabricated Fe_3_O_4_@MOF–Fe(II) core–shell particles were naturally cooled to room temperature and separated by an external magnet. For the washing process, Fe_3_O_4_@MOF–Fe(II) particles were sonicated three times in DMF, water, and ethanol for 15 min. The particles were vacuum dried at 110 °C overnight and labeled as Fe_3_O_4_@MOF–Fe(II).

### Synthesis of Fe_3_O_4_@HKUST-1

For the synthesis of Fe_3_O_4_@HKUST-1 core-shell particles with a magnetic core, Fe_3_O_4_–COOH nanoclusters were first dispersed in 100 mL of ethanol (5 mg/mL) under vigorous stirring for 30 min. Then, 0.343 g of Cu(NO_3_)_2_·3H_2_O was added and the suspension was sonicated for 1 h to facilitate copper metal ion coordination on the carboxylate groups of the Fe_3_O_4_–COOH cluster surface. The obtained solution was transferred to a 250 mL round bottom flask and heated at 85 °C using an external oil bath with a magnetic stirrer. After 1 h, 100 mL of H_3_BTC solution (3.61 mg/mL in ethanol) was added dropwise to the suspension and the mixture was stirred at 85 °C for a further 24 h to homogeneously grow the HKUST-1 shell. The fabricated Fe_3_O_4_@HKUST-1 core–shell particles were naturally cooled to room temperature and separated by an external magnet. For the washing process, Fe_3_O_4_@HKUST-1 particles were sonicated three times in ethanol and dichloromethane for 15 min. The particles were vacuum dried at 120 °C overnight and labeled as Fe_3_O_4_@HKUST-1.

### Synthesis of Fe_3_O_4_@UiO-66

For the synthesis of Fe_3_O_4_@UiO-66 core–shell particles with a magnetic core, Fe_3_O_4_–COOH nanoclusters were first dispersed in 100 mL of DMF (5 mg/mL) under vigorous stirring for 30 min. Then, 0.64 g of ZrCl_4_ was added and the suspension was sonicated for 1 h to facilitate zirconium metal ion coordination on the carboxylate groups of the Fe_3_O_4_–COOH cluster surface. The obtained solution was transferred to a 250 mL round bottom flask, mixed with 2 mL of AcOH, and heated at 120 °C using an external oil bath with a magnetic stirrer. After 1 h, 100 mL of H_2_BDC solution (4.56 mg/mL in DMF) was added dropwise to the suspension and the mixture was stirred at 120 °C for a further 24 h to homogeneously grow the UiO-66 shell. The fabricated Fe_3_O_4_@UiO-66 core–shell particles were naturally cooled to room temperature and separated by an external magnet. For the washing process, Fe_3_O_4_@UiO-66 particles were sonicated three times in hot DMF, water, and ethanol for 15 min. The particles were vacuum dried at 110 °C overnight and labeled as Fe_3_O_4_@UiO-66.

### Synthesis of Fe_3_O_4_@UiO-66–NH_2_

For the synthesis of Fe_3_O_4_@UiO-66–NH_2_ core-shell particles with a magnetic core, Fe_3_O_4_–COOH nanoclusters were first dispersed in 100 mL of DMF (5 mg/mL) under vigorous stirring for 30 min. Then, 0.64 g of ZrCl_4_ was added and the suspension was sonicated for 1 h to facilitate zirconium metal ion coordination on the carboxylate groups of the Fe_3_O_4_–COOH cluster surface. The obtained solution was transferred to a 250 mL round bottom flask, mixed with 2 mL of AcOH, and heated at 120 °C using an external oil bath with a magnetic stirrer. After 1 h, 100 mL of H_2_BDC solution (4.97 mg/mL in DMF) was added dropwise to the suspension and the mixture was stirred at 120 °C for a further 24 h to homogeneously grow the UiO-66–NH_2_ shell. The fabricated Fe_3_O_4_@UiO-66–NH_2_ core–shell particles were naturally cooled to room temperature and separated by an external magnet. For the washing process, Fe_3_O_4_@UiO-66–NH_2_ particles were sonicated three times in hot DMF, water, and ethanol for 15 min. The particles were vacuum dried at 110 °C overnight and labeled as Fe_3_O_4_@UiO-66–NH_2_.

### Synthesis of Fe_3_O_4_@MOF–SO_4_ nanomaterials

The pre-prepared core-shell materials were used to synthesize Fe_3_O_4_@MOF–SO_4_ nanocatalysts. Typically, 1 g of Fe_3_O_4_@MOF was dispersed in 500 mL of 0.05 M aqueous H_2_SO_4_ solution. After sonicating for 15 min, the solution was gently stirred for 24 h at room temperature. The nanocatalysts were washed three times to remove excess H_2_SO_4_ molecules. In each washing step, Fe_3_O_4_@MOF–SO_4_ were magnetically separated, dispersed in 250 mL ultra-pure hot water (ca. 60 °C), and sonicated for 15 min, followed by magnetic separation and supernatant removal. Finally, the products were dried using a vacuum oven at 150 °C for 48 h and stored for future use.

### Theoretical calculations

The initial structure of UiO-66 was taken from Cavka et al.^38^, and truncated and protonated based on the work of Sittiwong et al.^39^. This structure comprises four 1,4-benzene-dicarboxylate (BDC) linkers surrounding the defect active site on the Zr_6_O_4_(OH)_4_ node created by removing one linker, and seven linkers truncated to formate, for computational efficiency. To balance the charge of the system, hydrogens were added to the terminal carboxylates of BDC, and hydrogen was removed from the bridging OH of the defect active site to compensate for the removal of the BDC linker (Supplementary Fig. 3a). Following initial geometry optimization, a water molecule was added to the active site. The protonation of bridging oxygen and coordination of a hydroxyl group to a Zr atom was found to be more energetically favorable than an intact H_2_O by ~0.26 eV (Supplementary Fig. 3b). To model the effects of sulfation, an H_2_SO_4_ molecule was added to the hydroxylated defect active site, which spontaneously reacted with the adjacent hydroxyl group coordinated to the Zr to form HSO_4_^-^ coordinated to one Zr, and H_2_O coordinated to the other Zr, during geometry optimization (Supplementary Fig. 3c).

All DFT calculations were performed using Gaussian 16 Revision C.01^40^. The 6-31G(d,p) basis set was employed for all atoms except Zr, which was treated using the double-ζ of the Stuttgart–Dresden pseudopotential^41,42^ from the Basis Set Exchange^43^. The M06-L DFT functional^7^ was used for all calculations. For all geometry optimizations, the formate hydrogens and terminal carboxyl carbons were frozen to maintain the overall structure of UiO-66, while all other atoms were allowed to relax. This methodology has been validated previously for catalytic reactions on UiO-66^39^.

## Supplementary information

Supplementary Information

## Data Availability

The data that support the findings of this study are available from the corresponding authors upon reasonable request.
