# Supplementary material for: Engineered assembly of water-dispersible nanocatalysts enables low-cost and green CO2 capture
Source: Nat Commun. 2022 Mar 10;13:1249. doi: 10.1038/s41467-022-28869-6 (PMC8913730; doi:10.1038/s41467-022-28869-6)
Supplement: Supplementary file 1 — Supplementary Information [file 41467_2022_28869_MOESM1_ESM.pdf]

Supplementary Information

Engineered assembly of water-dispersible nanocatalysts enables low-cost and green CO_2 capture

Masood S. Alivand[1], Omid Mazaheri[1, 2], Yue Wu[1], Ali Zavabeti[1, 3], Andrew J. Christofferson[3, 4], Nastaran Meftahi[4], Salvy P. Russo[4], Geoffrey W. Stevens[1], Colin A. Scholes[1], Kathryn A. Mumford[1, *]

[1] Department of Chemical Engineering, The University of Melbourne, Melbourne, Victoria, 3010 Australia.

[2] School of Agriculture and Food, Faculty of Veterinary and Agricultural Sciences, The University of Melbourne, Melbourne, Victoria, 3010 Australia.

[3] School of Science, RMIT University, Melbourne, Victoria, 3001 Australia.

[4] ARC Centre of Excellence in Exciton Science, School of Science, RMIT University, Melbourne, Victoria, 3000 Australia.

* Corresponding author. Email: mumfordk@unimelb.edu.au; Fax: +61 3 83444153

Table of Contents

Supplementary Methods 3

Supplementary Note 1. General materials 3

Supplementary Note 2. Characterization 3

Supplementary Note 3. Hammett indicator test 5

Supplementary Note 4. Synthesis of heterogenous nanomaterials 5

Supplementary Note 5. CO_2 capture experiments 9

Supplementary Note 6. Theoretical calculations 14

Supplementary Discussion 15

Supplementary References 48

Supplementary Methods

Supplementary Note 1. General materials

Iron (III) chloride hexahydrate ($FeCl_3.6H_2O$, >98.0%), iron (II) chloride tetrahydrate ($FeCl_2.4H_2O$, 98.0%), iron (II) sulfate heptahydrate ($FeSO_4.7H_2O$, >99.0%), ferric ammonium citrate (reagent grade), zirconium (IV) chloride ($ZrCl_4$, >99.5%), zirconyl chloride octahydrate ($ZrOCl_2.8H_2O$, 98.0%), zinc nitrate hexahydrate ($Zn(NO_3)_2 \cdot 6H_2O$, 98.0%), cobalt (II) nitrate hexahydrate ($Co(NO_3)_2 \cdot 6H_2O$, >98.0%), copper (II) nitrate trihydrate ($Cu(NO_3)_2 \cdot 3H_2O$, >99.0%), benzene-1,4-dicarboxylic acid (H_2BDC, 98.0%), 2-aminoterephthalic acid (H_2BDC-NH_2, 99.0%), benzene-1,3,5-tricarboxylic acid (H_3BTC, 95.0%), 2,5-pyridinedicarboxylic acid (H_2BDC-N, 98%), 2-methylimidazole (2-MeIm, 99%), 3-(N-Morpholino)propanesulfonic acid (MOPS, >99.5%), 1,3-Bis[tris(hydroxymethyl)methylamino] propane (BIS-TRIS, >99.0%), n, n-dimethylformamide (DMF, >99.8%), urea (>99.0%), ammonium hydroxide solution (NH_4OH, 28-30%) and all Hammett indicators were purchased from Sigma-Aldrich and used as received. Sodium acetate (NaOAc, >99.0%), monoethanolamine (MEA, >99.5%), ethylene glycol (EG, >99.8%), methanol (>99.0%), ethanol (>99.5%), dichloromethane (>99.5%), hydrochloric acid (HCl, 32.0%), sulfuric acid (H_2SO_4, 98.5%), acetic acid (AcOH, 99.8%) were purchased from Chem-Supply, Australia. Ultra-pure nitrogen (N_2, 99.9%) and carbon dioxide (CO_2, 99.9%) were supplied by BOC Gases Australia and used for CO_2 absorption-desorption experiments.

Supplementary Note 2. Characterization

Raman spectroscopy was carried out on a Renishaw inVia Qontor confocal Raman microscope equipped with a 532 nm laser source. Before each test, the samples were dispersed in ethanol, coated on a silicon wafer and dried at room temperature under vacuum. X-ray photoelectron

spectroscopy (XPS) was conducted on a VG ESCALAB 220i-XL spectrometer with A1 Kα radiation and 1486.6 eV photon energy. During the characterization, the X-ray anode and chamber pressure were kept at 220 W (22 mA, 10 kV and 45˚ detection angle) and 10^{-7} Pa absolute pressure, respectively. The broad survey and high-resolution scans were acquired with 1.0 and 0.05 eV resolution, respectively. CasaXPS processing software was used to analyze the obtained data. Attenuated total reflectance Fourier transform infrared (ATR-FTIR) spectra were measured using a Bruker Tensor II. For each spectrum, 64 scans were recorded in 400-4000 cm^{-1} range with 4 cm^{-1} resolution. X-ray diffraction (XRD) patterns were acquired using Bruker D8 Advance instrument with Cu Kα radiation (40 mA and 40 kV). Thermogravimetric analysis (TGA) was performed on a NETZSCH TG 209 F1 Libra analyzer in 30-800 ˚C range with 10 ˚C/min heating rate in N_2 atmosphere. The Brunauer-Emmett-Teller (BET) specific surface area, pore volume and pore size distribution of the materials were analyzed using nitrogen adsorption-desorption technique at -196 ˚C on a Micrometrics 3Flex instrument. Before each analysis, the materials were degassed at 140 ˚C under vacuum pressure overnight to remove undesired adsorbed gas molecules. Zeta potential and thermal conductivity were measured by a Malvern Zetasizer Nano ZS and NETZSCH HyperFlash LFA467, respectively. Scanning electron microscopy (SEM) was performed on a FEI Teneo instrument with an operating voltage of 30 kV. Before taking SEM images, the materials were sputtered with 10 nm gold by a Quorum K575X ion sputter instrument with 30 mA current. For the preparation of TEM samples, the nanomaterials were first dispersed in ethanol (0.01wt.% solution), then a single droplet was directly dropped onto the formvar carbon-coated copper TEM grid and used for TEM imaging. Helium ion microscopy (HIM) images were captured on a Zeiss Orion NanoFab microscope, operating at 25 kV. Transmission electron microscopy (TEM), energy-dispersive X-ray spectroscopy (EDX) mapping and line spectra analysis were carried out on a JEOL 2100f instrument with a 200 kV acceleration voltage, equipped with oxford X-Maxn

80T EDS detector Gatan OneView 4k camera. The elemental analysis was conducted on a Perkin Elmer CHNS Analyzer, 2400 Series II.

Supplementary Note 3. Hammett indicator test

For the preparation of Hammett indicator solutions with a constant 0.5 wt.% concentration, a weighed amount of each Hammett indicator was dissolved in anhydrous benzene in an inert atmosphere glovebox (99.9% nitrogen). Then, 50 mg of each water-dispersible nanocatalyst was dispersed in 5 mL of each Hammett indicator solution and stirred for 30 min. The dispersed nanomaterials were separated using an external neodymium magnet and the color of the solution was recorded. The details of each Hammett indicator used are presented in Supplementary Table 1.

Supplementary Note 4. Synthesis of heterogenous nanomaterials

Conventional Fe_3O_4 nanoparticles. To compare the structural properties of Fe_3O_4-COOH nanoclusters with a benchmark, conventional Fe_3O_4 nanoparticles were synthesized according to the previously reported co-precipitation method with minor modifications[1]. Firstly, 298.5 mg of $FeCl_2.4H_2O$ and 810 mg of $FeCl_3.6H_2O$ were dissolved in 100 mL of water and sonicated for 15 min. The temperature was increased to 60 ˚C, and while keeping the mixture under vigorous agitation, NH_4OH solution was added as an oxidation agent to trigger the iron oxide precipitation. When the pH of solvent reached 11, the temperature was increased to 80 ˚C and held for 2 hr. The black precipitates were separated by an external magnet and washed with a copious amount of water to remove extra NH_4OH reactants and OH^- ions. Eventually, the obtained products were dried in a vacuum oven at 80 ˚C overnight, labelled as Fe_3O_4 nanoparticles and stored for future use.

$SO_4^{2-}/ZrO_2/Al_2O_3$. The $SO_4^{2-}/ZrO_2/Al_2O_3$ catalyst was synthesized according to previous literature with minor modifications[2]. Briefly, Al_2O_3 (7.0 g) was dispersed in water (120 mL) followed by adding $ZrOCl_2.8H_2O$ (9.35 g) and urea (50 g). The resulting mixture was sonicated for 30 min and stirred at 90 ˚C for 5 hr. At this stage, the products were filtered, washed with copious amount of water and dried at 90 ˚C overnight. Then, the solid materials were calcinated at 550 ˚C in air atmosphere for 6 hr with a rate of temperature increase of 5 ˚C/min and denoted as ZrO_2/Al_2O_3. To sulfate the structure, the obtained materials were subjected to aqueous H_2SO_4 solution (0.7 M, 15 mL solution for each 1 g sample) and stirred at room temperature for 3 hr. Finally, the materials were filtered, dried at 110 ˚C overnight and calcinated in air atmosphere (550 ˚C, 6 hr) with a rate of temperature increase of 5 ˚C/min. The prepared materials were labelled as $SO_4^{2-}/ZrO_2/Al_2O_3$.

SO_4^{2-}/ZrO_2/SBA-15. The SO_4^{2-}/ZrO_2/SBA-15 catalyst was synthesized according to previous literature with minor modifications[3]. Briefly, SBA-15 (7.0 g) was dispersed in water (120 mL) followed by adding $ZrOCl_2.8H_2O$ (9.35 g) and urea (50 g). The resulting mixture was sonicated for 30 min and stirred at 90 ˚C for 5 hr. At this stage, the products were filtered, washed with copious amount of water and dried at 90 ˚C overnight. Then, the solid materials were calcinated at 550 ˚C in air atmosphere for 6 hr with a rate of temperature increase of 5 ˚C/min and denoted as ZrO_2/SBA-15. To sulfate the structure, the obtained materials were subjected to aqueous H_2SO_4 solution (0.7 M, 15 mL solution for each 1 g sample) and stirred at room temperature for 3 hr. Finally, the materials were filtered, dried at 110 ˚C overnight and calcinated in air atmosphere (550 ˚C, 6 hr) with a rate of temperature increase of 5 ˚C/min. The prepared materials were labelled as SO_4^{2-}/ZrO_2/SBA-15.

ZIF-8. The ZIF-8 crystals were synthesized according to previous literature[4]. Briefly, 2-MeIm (1.41 g) and $Zn(NO_3)_2 \cdot 6H_2O$ (1.62 g) were separately dissolved in 100 mL of methanol under

sonication. Then, the methanolic solution of zinc was gradually added to the 2-MeIm solution and the mixture stirred for 24 hr at room temperature. The white crystals of ZIF-8 were separated by centrifugation (12,000 rcf, 10 min) and washed three times by methanol. The final product was vacuum dried at 150 ˚C overnight and labeled as ZIF-8.

ZIF-67. The ZIF-67 crystals were synthesized according to previous literature with minor modifications[5]. Briefly, 2-MeIm (1.12 g) and $Co(NO_3)_2 \cdot 6H_2O$ (1.00 g) were separately dissolved in 80 mL of methanol under ultrasonication. Then, the methanolic solution of cobalt was gradually added to the 2-MeIm solution and the mixture was stirred for 24 hr at room temperature. The ZIF-67 crystals were separated by centrifugation (12,000 rcf, 20 min) and washed three times by methanol. The final product was vacuum dried at 150 ˚C overnight and labeled as ZIF-67.

MIL-100(Fe). The MIL-100(Fe) crystals were synthesized according to previous literature with minor modifications[6]. Briefly, H_3BTC (3.44 g) and $FeCl_3 \cdot 6H_2O$ (1.4 g) were separately dissolved in 100 mL of water under sonication. Then, the aqueous solution of iron was gradually added to the H_3BTC solution, mixed with 2 mL of HF acid and stirred for 1 hr. The obtained solution was transferred to a stainless-steel autoclave (150 mL capacity) and heated at 160 ˚C for 12 hr. The crystals of MIL-100(Fe) were separated by centrifugation (12,000 rcf, 10 min) and washed three times by water. The final product was vacuum dried at 110 ˚C overnight and labeled as MIL-100(Fe).

MOF-Fe(II). The MOF-Fe(II) crystals were synthesized according to our previous literature with minor modifications[7]. Briefly, H_2BDC-N (1.67 g) and $FeSO_4$ (2.78 g) were respectively dissolved in 100 mL of DMF and water under sonication. Then, the aqueous solution of iron was gradually added to the H_2BDC-N solution and stirred for 1 hr. The obtained solution was transferred to a 250 mL round bottom flask and heated at 130 ˚C for 4 hr using an external oil

bath with magnetic stirrer. After gradually cooling to room temperature, the orange crystals of MOF-Fe(II) were separated by centrifugation (8000 rcf, 15 min) and washed three times by DMF, water and ethanol. The final product was vacuum dried at 110 ˚C overnight and labeled as MOF-Fe(II).

HKUST-1. The crystals of HKUST-1 were synthesized according to previous literature with minor modifications[8]. Briefly, H_3BTC (2.1 g) and $Cu(NO_3)_2 \cdot 3H_2O$ (2.0 g) were separately dissolved in 80 mL ethanol and 50:50 vol.% water/DMF mixture under sonication. Then, the ethanolic solution of H_3BTC was gradually added to the copper solution and stirred for 1 hr. The obtained solution was transferred to a 250 mL round bottom flask and heated at 85 ˚C for 24 hr using an external oil bath with magnetic stirrer. The crystals of HKUST-1 were separated by centrifugation (12,000 rcf, 20 min) and washed three times by dichloromethane. The final product was vacuum dried at 170 ˚C overnight and labeled as HKUST-1.

UiO-66. The crystals of UiO-66 were synthesized according to previous literature with minor modifications[9]. Briefly, H_2BDC (1.368 g) and $ZrCl_4$ (1.920 g) were separately dissolved in 60 mL of DMF under ultrasonication. Then, the zirconium solution was mixed with 12 mL of AcOH acid, gradually added to the H_2BDC solution and stirred for 1 hr. The obtained solution was transferred to a 250 mL round bottom flask and heated at 120 ˚C for 24 hr using an external oil bath with magnetic stirrer. The white crystals of UiO-66 were separated by centrifugation (12,000 rcf, 10 min) and washed three times by hot DMF, water and ethanol. The final product was vacuum dried at 110 ˚C overnight and labeled as UiO-66.

UiO-66-NH_2. The crystals of UiO-66-NH_2 were synthesized according to previous literature with minor modifications[10]. Briefly, H_2BDC-NH_2 (1.491 g) and $ZrCl_4$ (1.92 g) were separately dissolved in 60 mL of DMF under ultrasonication. Then, the zirconium solution was gradually added to the H_2BDC-NH_2 solution, mixed with 12 mL of AcOH acid and stirred for 1 hr. The

obtained solution was transferred to a 250 mL round bottom flask and heated at 120 ˚C for 24 hr using an external oil bath with magnetic stirrer. The yellow crystals of UiO-66-NH_2 were separated by centrifugation (12,000 rcf, 10 min) and washed three times by hot DMF, water and ethanol. The final product was vacuum dried at 110 ˚C overnight and labeled as UiO-66-NH_2.

Supplementary Note 5. CO_2 capture experiments

Cyclic CO_2 absorption-desorption. Both CO_2 absorption and desorption experiments were conducted in an in-house modified OptiMax™ workstation 1001 (Mettler-Toledo) connected to a dynamic gas flow apparatus (Supplementary Figure 1)[11,12]. The device was equipped with a 1000 mL reactor, an adjustable mixer to keep the solution uniform, and a temperature controller system including a thermocouple and a heating jacket vessel to accurately control the reactor operating temperature and measure heat transfer parameters.

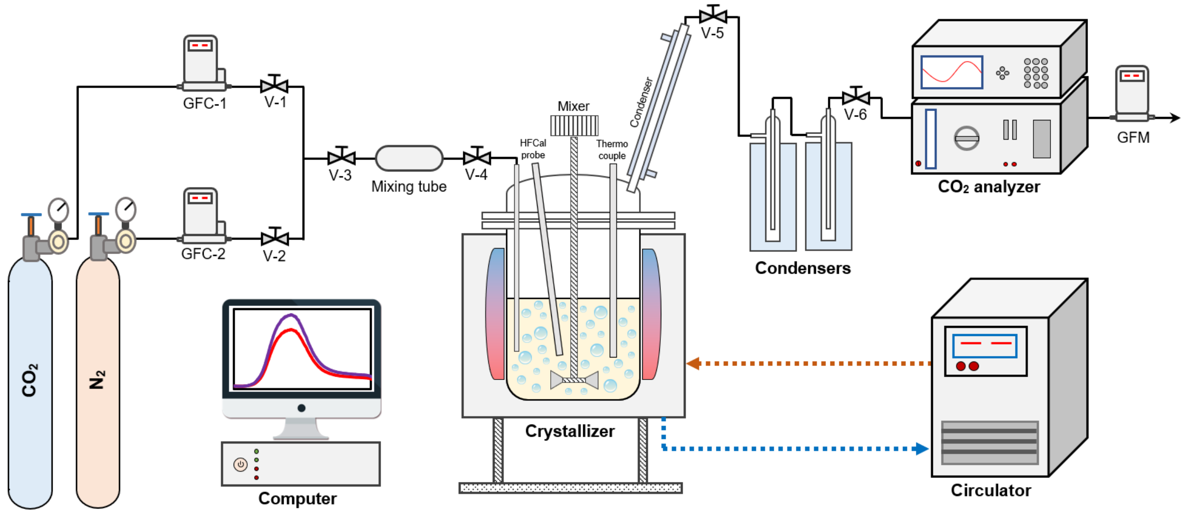

Supplementary Figure 1. Schematic diagram of the CO_2 absorption-desorption apparatus operating at 40-88 ˚C and atmospheric pressure.

In a typical CO_2 absorption experiment, 500 mL of pre-prepared aqueous 5M MEA solution (nearly equivalent to 30 wt.% MEA in water) and a desired amount of catalyst (varied from 0.01 to 0.1 wt.%) was mixed. It should be noted that no catalyst was added at this stage for the blank experiments. The prepared solution was sonicated for 30 min at 60 ˚C and then transferred to the reactor. To mimic the operating conditions of CO_2 absorption from post-combustion flue gas streams, the reactor temperature, pressure and rotation speed were set at 40 ˚C, 40 kPa and 400 rpm, respectively. Then, a constant flow of a CO_2/N_2 binary mixture (635 mL/min, 15 vol.% CO_2 and 85 vol.% N_2) was bubbled into the solvent. For this purpose, two separate gas flow controllers were utilized to provide constant CO_2 (Aalborg, CO_2-GFC17, 0-100 mL/min) and N_2 (Aalborg, N_2-GFC17, 0-10 L/min) streams. The treated gas stream was cooled using a Graham condenser connected to an external water circulator (-2±0.1 ˚C) and the evaporated solvent returned to the reactor. Then, it passed through two consecutive ice bath condensers (acetone-water mixture with -15±5 ˚C) to ensure any remaining moisture was trapped. The concentration of CO_2 and the volumetric flow rate of the treated gas stream were measured using an online CO_2 analyzer (BlueSens, BCP-CO_2) and a digital flow meter (Aalborg, GFM17, 0-1000 mL/min), respectively. During the CO_2 absorption experiment, the concentration of CO_2 at the outlet stream was regularly monitored and the binary gas flow stopped bubbling when the concentration of CO_2 at outlet stream reached 15±0.1 vol.%.

For CO_2 desorption experiment, CO_2 gas flow stopped and N_2 gas flow continued as the carrier gas with 90 mL/min flow rate. The CO_2 desorption process started by increasing the reactor temperature from 40 to 88 ˚C, maintained at this temperature for 30 min and finished by returning the temperature to 40 ˚C. To quantitatively perform the heat flow calorimetry analysis and measure the amount of energy consumption during the catalyst-aided solvent regeneration, OptiMax™ workstation was connected to OptiMax HFCal (Mettler-Toledo) probe. iControl software was used to directly record and evaluate all received information from OptiMax™

workstation and HFCal. Once the solvent regeneration finished, the reactor operating temperature was reduced to 40 ˚C and the N_2 inlet valve closed. The reactor was maintained at this temperature for taking samples and initiating the next CO_2 absorption-desorption cycle.

Reliability of measurements. To test the reliability of data measured by the online CO_2 analyzer, the CO_2 loading of the aqueous amine solution was also measured by a CO_2 Coulometer equipment (CM5015) with ±0.01 mol/L accuracy connected to an internal acidification module (CM5230). For each measurement, 2 mL CO_2-loaded amine solution was titrated using 1 mL concentrated H_2SO_4 to release its captured CO_2 molecules. The comparative results of CO_2 loading using gas and liquid measurement techniques are demonstrated in Supplementary Figure 2 confirming the reliability of quantitative CO_2 absorption-desorption measurement technique used in this study.

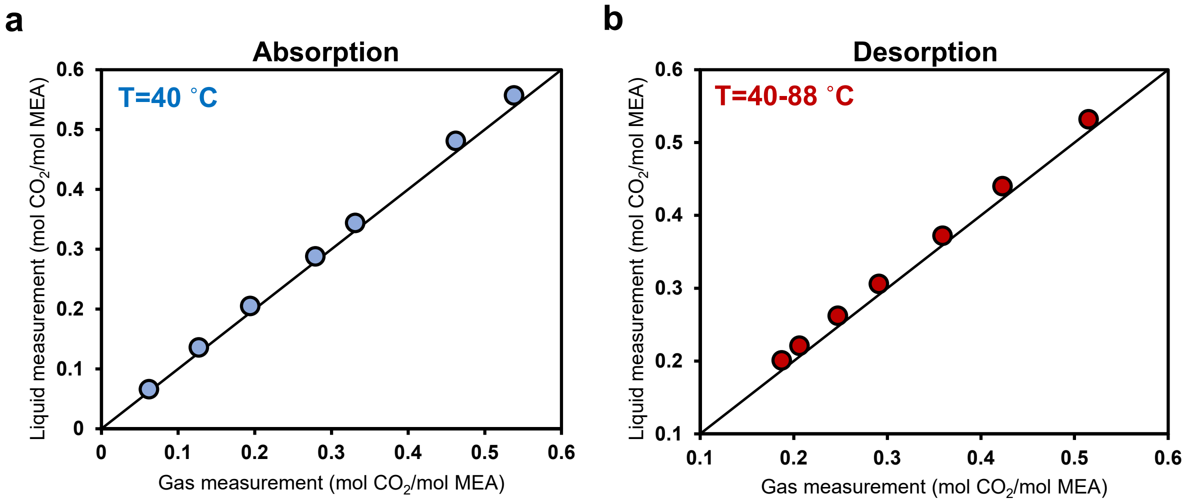

Supplementary Figure 2. Comparing the CO_2 loading of the aqueous 5 M MEA solution recorded using liquid and gas measurement methods during (a) absorption (blue dots) and (b) desorption (red dots) experiments. The operating temperature was kept constant at 40 ˚C during the CO_2 absorption, while it increased from 40 to 88 ˚C during solvent regeneration operation (CO_2 desorption).

Desorption calculations. The flow rate of desorbed CO_2 was calculated using the following equation (Supplementary Equation 1):

$$n_{CO_2} = \frac{X_{CO_2}}{1 - X_{CO_2}} n_{N_2} \tag{1}$$

where n_{CO_2} (mol/min) is the flow rate of CO_2 at outlet stream, n_{N_2} (mol/min) is the rate of N_2 at outlet stream and in the volume fraction of CO_2 in CO_2/N_2 binary mixture detected by the CO_2 analyzer.

The total amount of released CO_2 (N_{CO_2}, mol) during the solvent regeneration process (t, sec) was calculated by the following equation (Supplementary Equation 2):

$$N_{CO_2} = \int_0^t n_{CO_2}\, dt \tag{2}$$

The heat duty (HD, kJ/mol) of CO_2 desorption operation was calculated by the following equation (Supplementary Equation 3):

$$HD = \frac{E}{N_{CO_2}} \tag{3}$$

where E (kJ) is amount of consumed energy calculated according to the following equation (Supplementary Equation 4):

$$E = \int_0^t HF\, dt \tag{4}$$

where HF (kW) is the heat flow measured by HFCal probe.

To compare the efficiency of different heterogeneous catalysts, relative heat duty (RH, %) was calculated by the following equation (Supplementary Equation 5):

$$RH = \frac{HD_{Cat}}{HD_{Blank}} \times 100 \tag{5}$$

where HD_{Cat} (kJ) is the heat duty required for the solvent regeneration process in the presence of catalyst and HD_{Blank} (kJ) is the amount of required energy for the regeneration of the blank solvent without any catalysts.

The cyclic CO_2 absorption-desorption capacity (mol CO_2/mol amine) was calculated by Supplementary Equation 6 to present the efficiency of catalyst-aided solvent regeneration from a different point of view.

$$Cyclic\ capacity = \frac{C_{Rich} - C_{Lean}}{C_{MEA}} \quad (6)$$

where C_{Rich} (mol/L) and C_{Lean} (mol/L) are CO_2 loading into the solution before and after regeneration process, respectively, and C_{MEA} (mol/L) is the amine concentration in the aqueous solvent.

Supplementary Note 6. Theoretical calculations

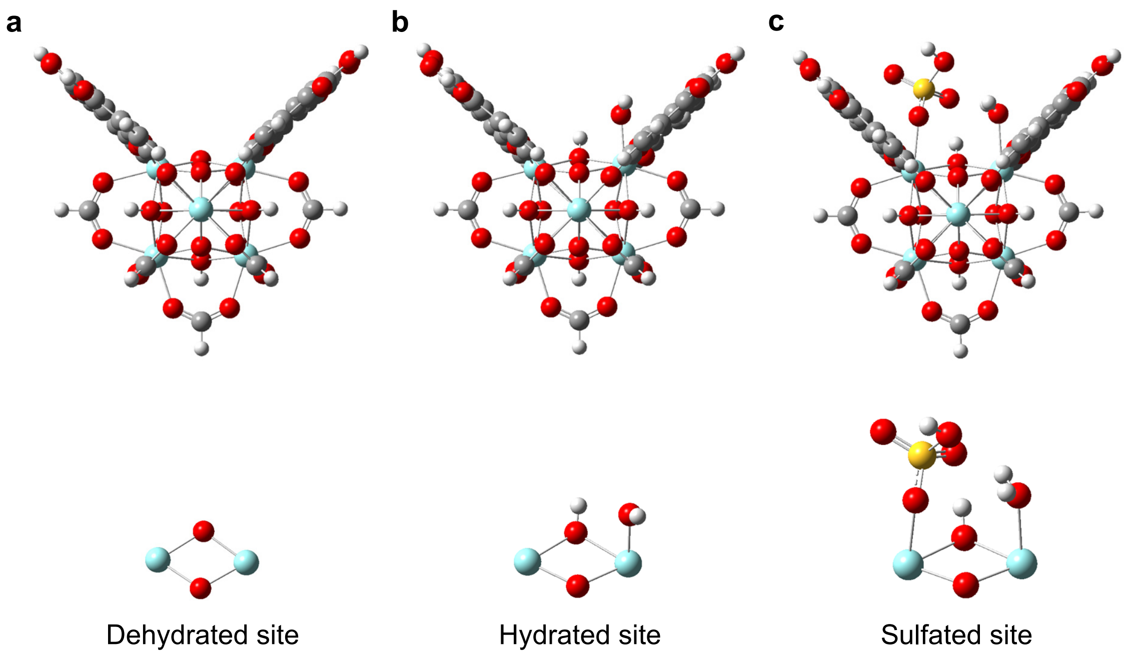

Supplementary Figure 3. Initial fragment structures of UiO-66. **(**a) Dehydrated defect active site. (b) Hydrated defect active site. (c) Sulfated active site. The full fragment is shown in the upper panel, and a rotated view of the defect active site alone is shown in the lower panel. Zirconium, sulfur, oxygen, carbon, and hydrogen are coloured cyan, yellow, red, grey, and white, respectively.

Supplementary Discussion

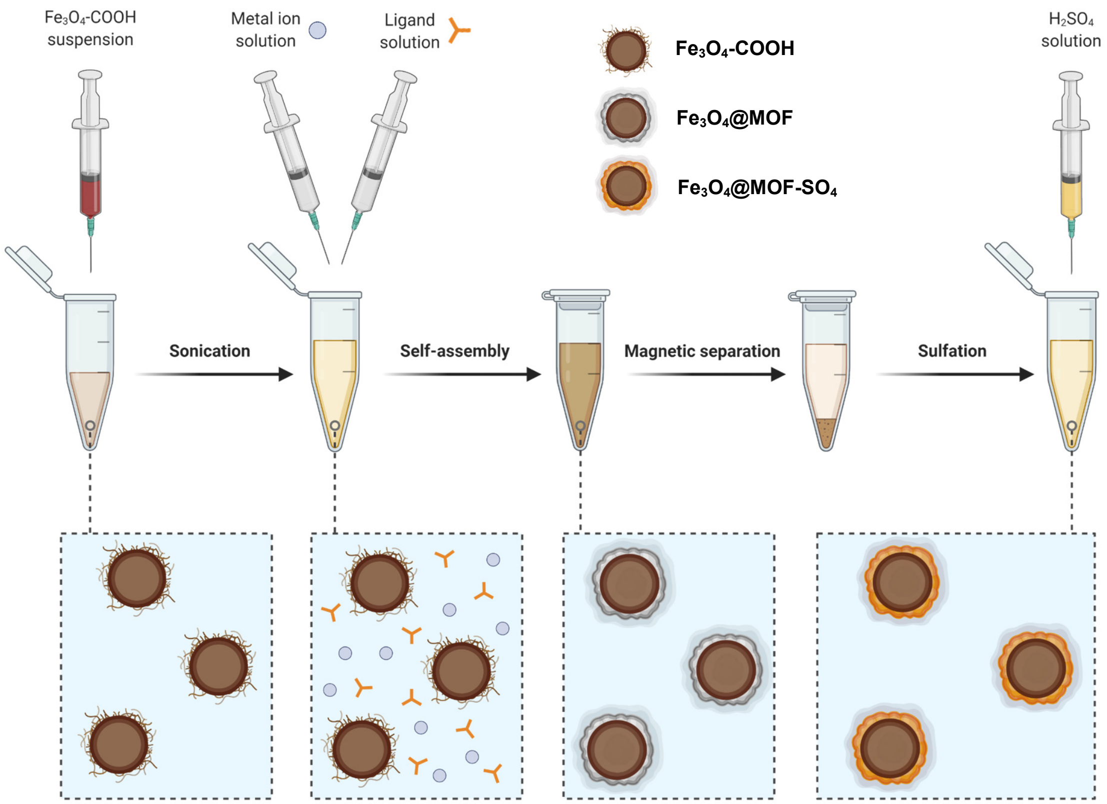

Supplementary Figure 4. Schematic illustration of the modulated self-assembly protocol used to prepare engineered Fe_3O_4@MOF and Fe_3O_4@MOF-SO_4 core-shell nanomaterials. The carboxylic acid groups implanted on the rough surface of magnetic nanoclusters could potentially interfere the conventional mechanism of MOF self-assembly and induce structural defects though the network of coated MOFs. Thus, this methodology enables the engineering of different MOF structures (different chemical and textural properties), creating hierarchical micro-mesoporous MOFs. The obtained core-shell materials can be easily sulfated owing to their defected networks and uncoordinated metal clusters. The sulfation process was performed in aqueous H_2SO_4 solution (0.05 M, room temperature) for 24 hr.

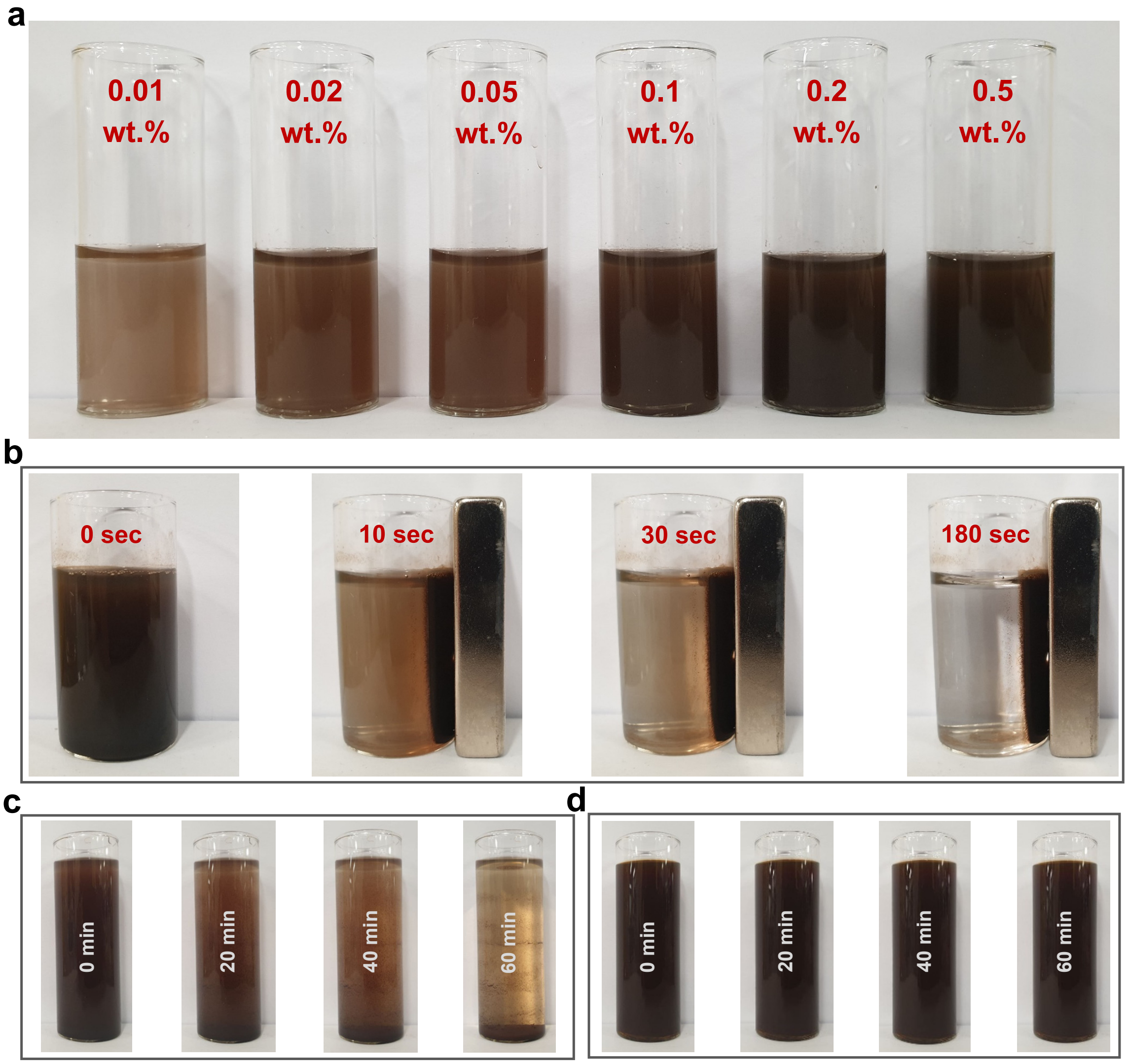

Supplementary Figure 5. (a) Photographs of water-dispersible Fe_3O_4-COOH nanoclusters at different concentrations in 0.01-0.5 wt.%. It is worth mentioning that magnetic nanoclusters remained stable in the aqueous solution (MOPS buffer solution, pH 7.5) for at least 72 hr. Before taking the images, all nanofluids were ultrasonicated for 15 min, then incubated by 6 hr at room conditions. (b) Photographs of Fe_3O_4-COOH separation at different time intervals in the presence of external neodymium magnet, indicating the ability of Fe_3O_4-COOH nanoclusters to be simply separated after its lifetime as an important matter for environmental concerns. Photographs of (c) conventional Fe_3O_4 nanoparticles and (d) Fe_3O_4-COOH nanoclusters stability versus time.

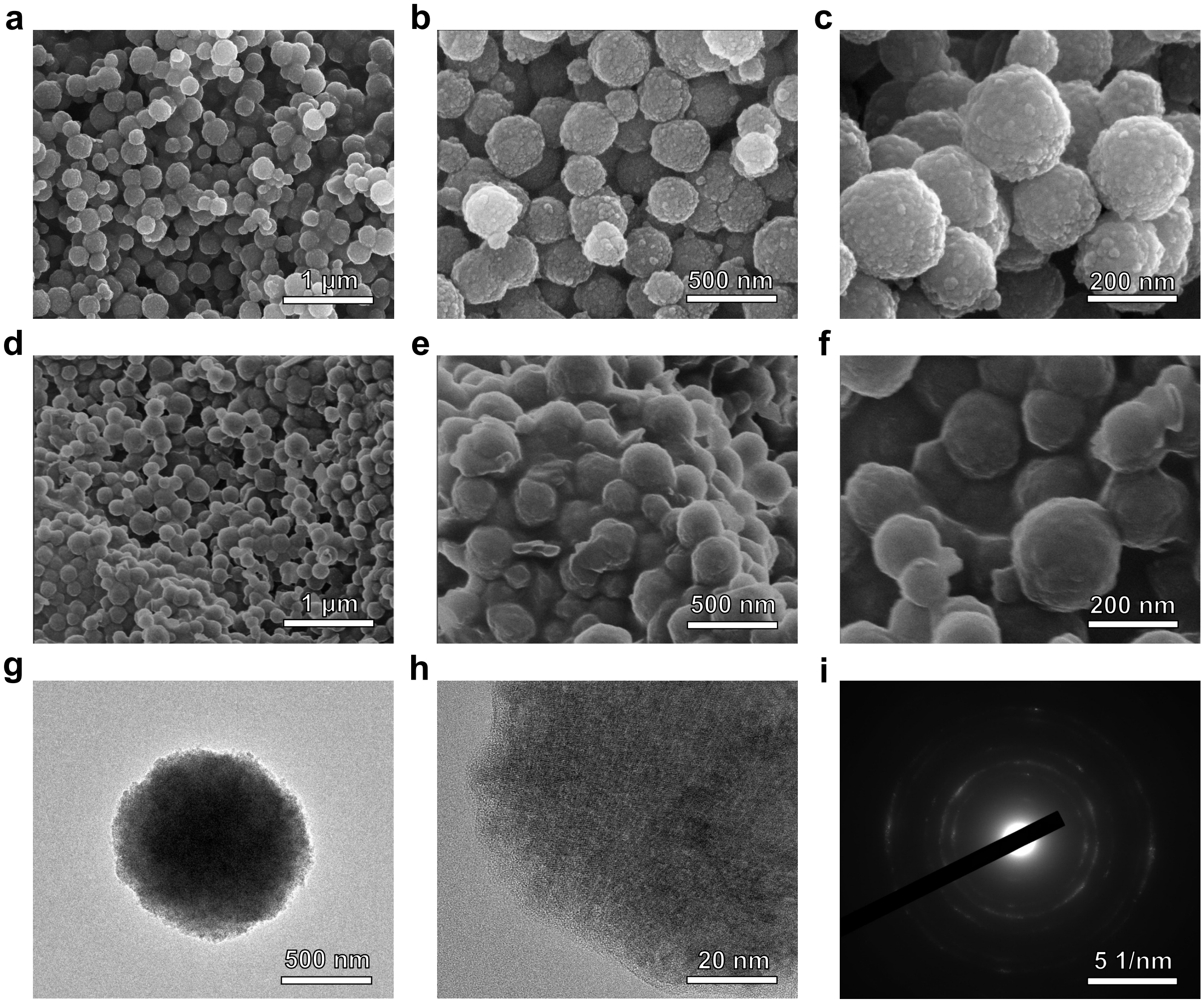

Supplementary Figure 6. Representative microscopy images of Fe_3O_4-COOH nanoclusters: (a-c) SEM and (d-f) HIM images, showing homogeneous and uniform nanoclusters with an average diameter of ~100-300 nm. (g-h) TEM and (i) selected area electron diffraction (SAED) images.

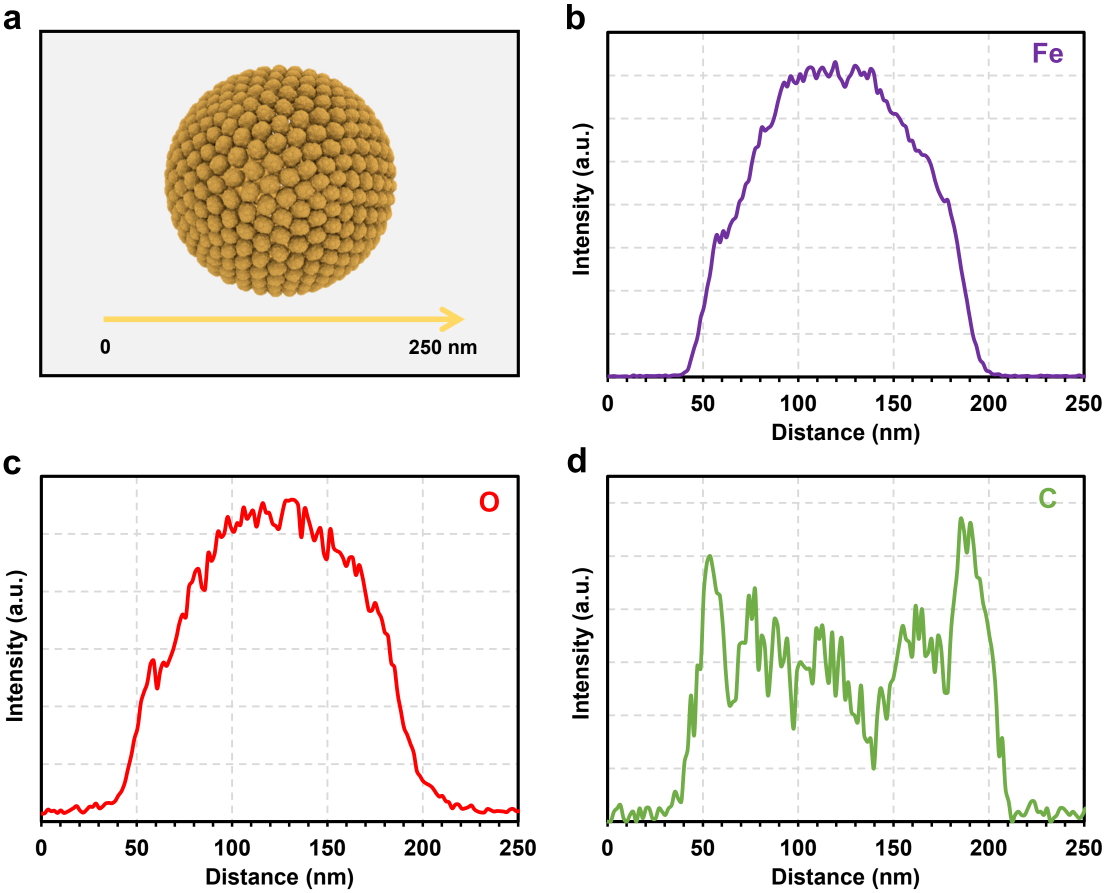

Supplementary Figure 7. (a) Schematic image and (b-d) elemental line scanning profiles (Fe, O and C) of water dispersible Fe_3O_4-COOH core-shell material. The presence of C on the surface of nanocluster is attributed to the carboxylic acid groups, homogeneously distributed on the exterior surface.

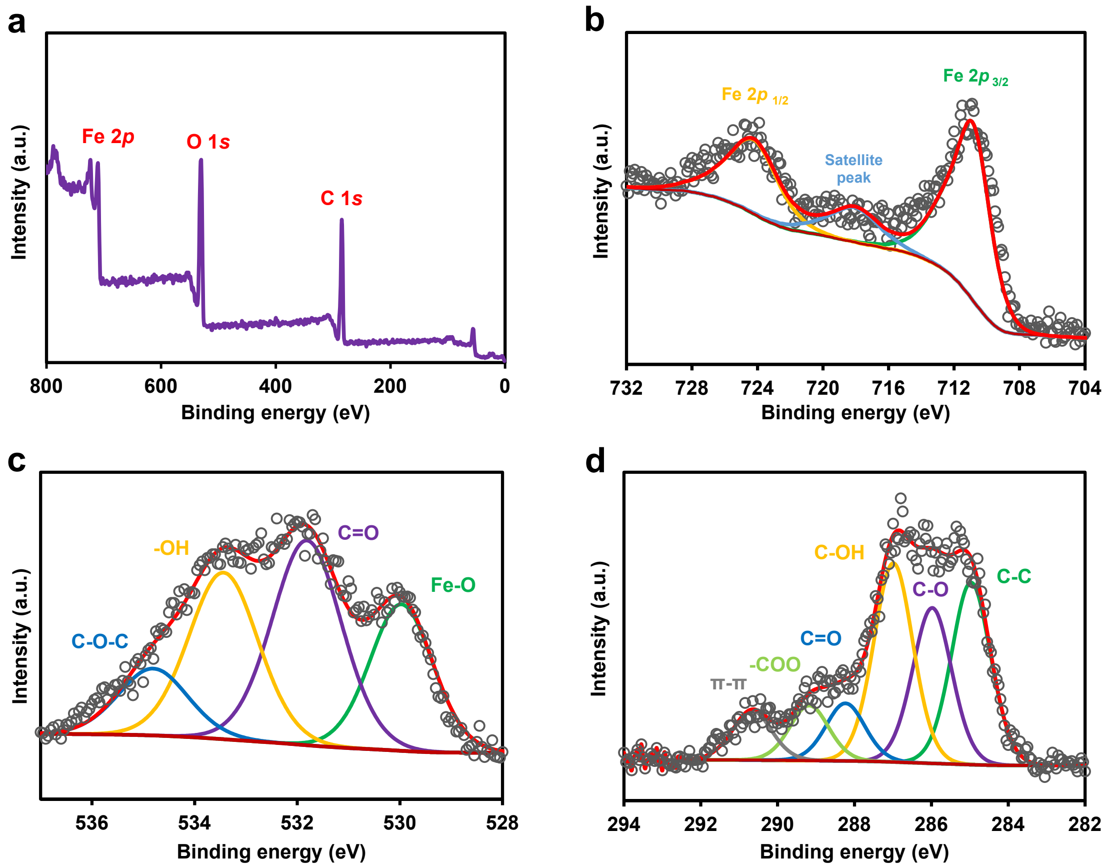

Supplementary Figure 8. XPS spectra of water-dispersible Fe_3O_4-COOH nanoclusters: (a) full survey atomic elemental distribution on the surface, high-resolution (b) Fe 2*p*, (c) O 1*s*, and (d) C 1*s*.

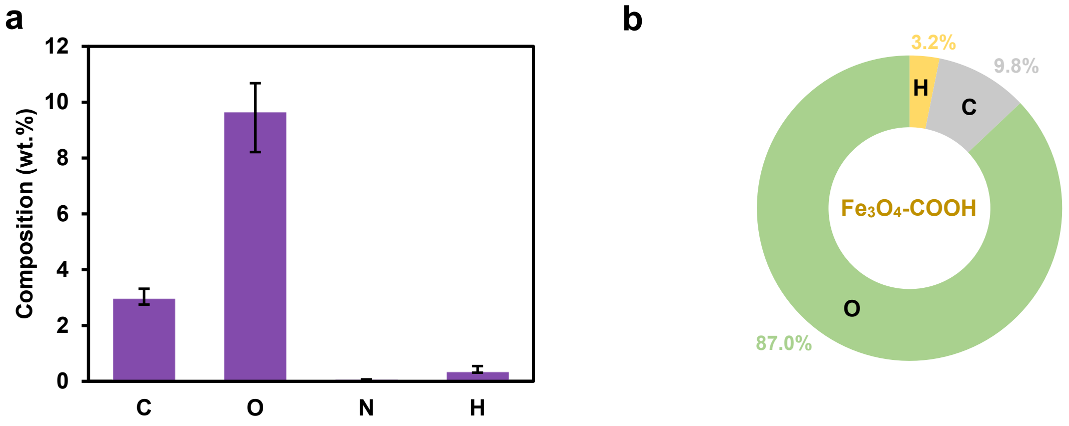

Supplementary Figure 9. Bulk elemental content of Fe_3O_4-COOH nanoclusters (a) with and (b) without considering Fe element. The results indicate the presence of both C and H elements, conforming the successful assembly of carboxylic acid groups on the surface.

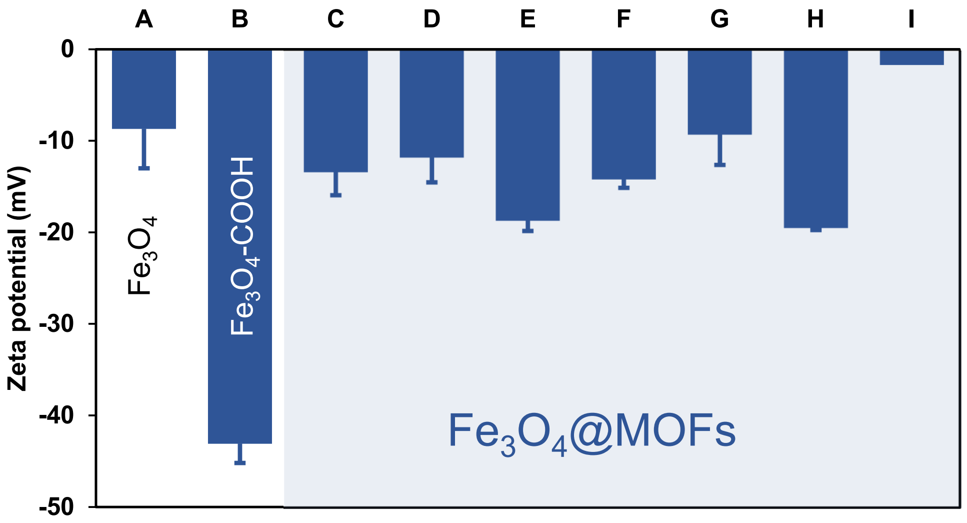

Supplementary Figure 10. Zeta potential values of conventional Fe_3O_4 nanoparticles (A), acidic Fe_3O_4-COOH nanoclusters (B) and Fe_3O_4@MOF core-shell nanomaterials. MOF: ZIF-8 (C), ZIF-67 (D), MOF-Fe(II) (E), MIL-100(Fe) (F), HKUST-1 (G), UiO-66 (H), and UiO-66-NH_2 (I). All the nanomaterials were dispersed in BIS-TRIS buffer solution (pH 9.0) and the concentration of materials was kept constant at 0.01 wt.%.

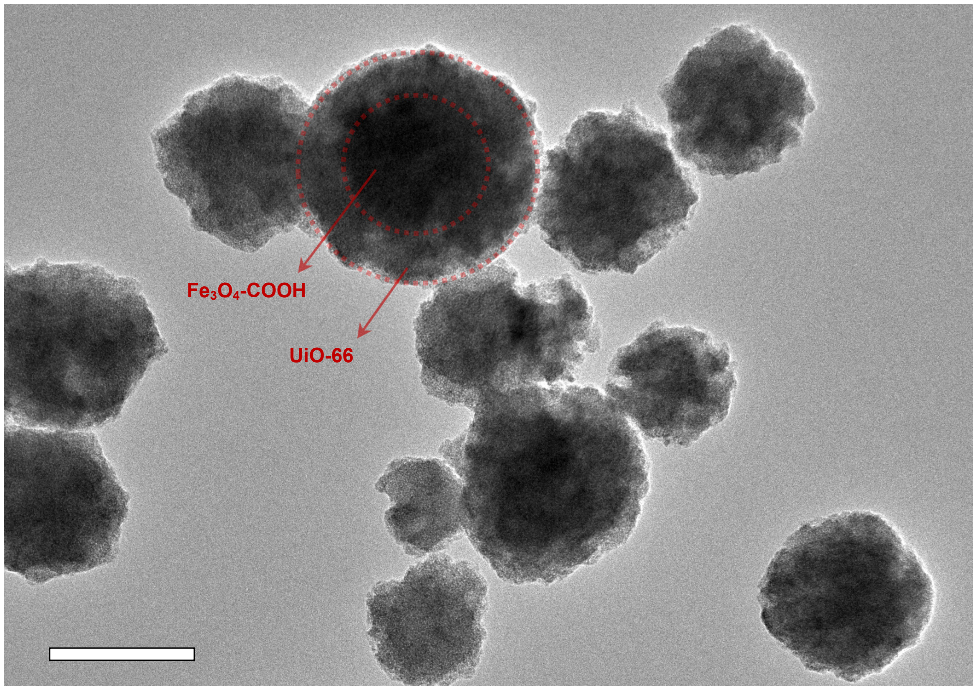

Supplementary Figure 11. Low-magnification TEM image of Fe_3O_4@UiO-66 core-shell nanomaterials. It can be seen that the obtained core-shell structures are uniform and MOF has evenly grown on the external surface of Fe_3O_4-COOH nanocluster. Scale bar is 200 nm.

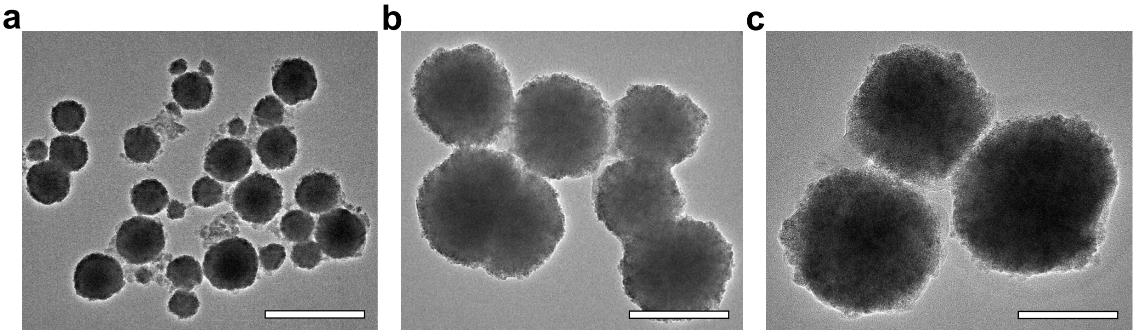

Supplementary Figure 12. TEM images of Fe_3O_4@HKUST-1 core-shell nanomaterials formed by modulated self-assembly of MOF on the carboxylate-rich surface of Fe_3O_4-COOH nanoclusters. Scale bars are (a) 500 nm, (b) 200 nm and (c) 100 nm.

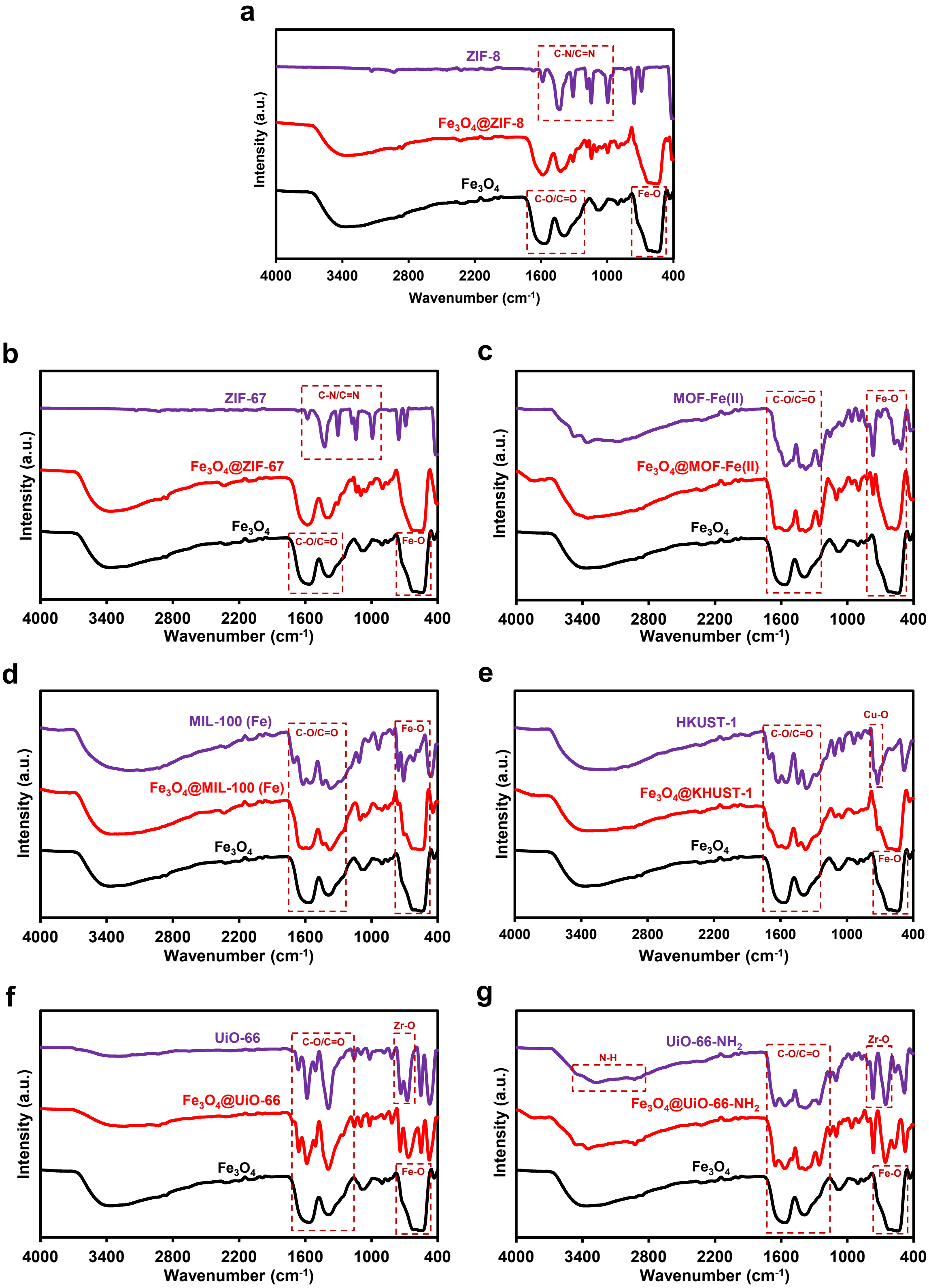

Supplementary Figure 13. FTIR analysis of pristine MOFs and core-shell nanomaterials: (a) ZIF-8, (b) ZIF-67, (c) MOF-Fe(II), (d) MIL-100(Fe), (e) HKUST-1, (f) UiO-66, and (g) UiO-66-NH_2.

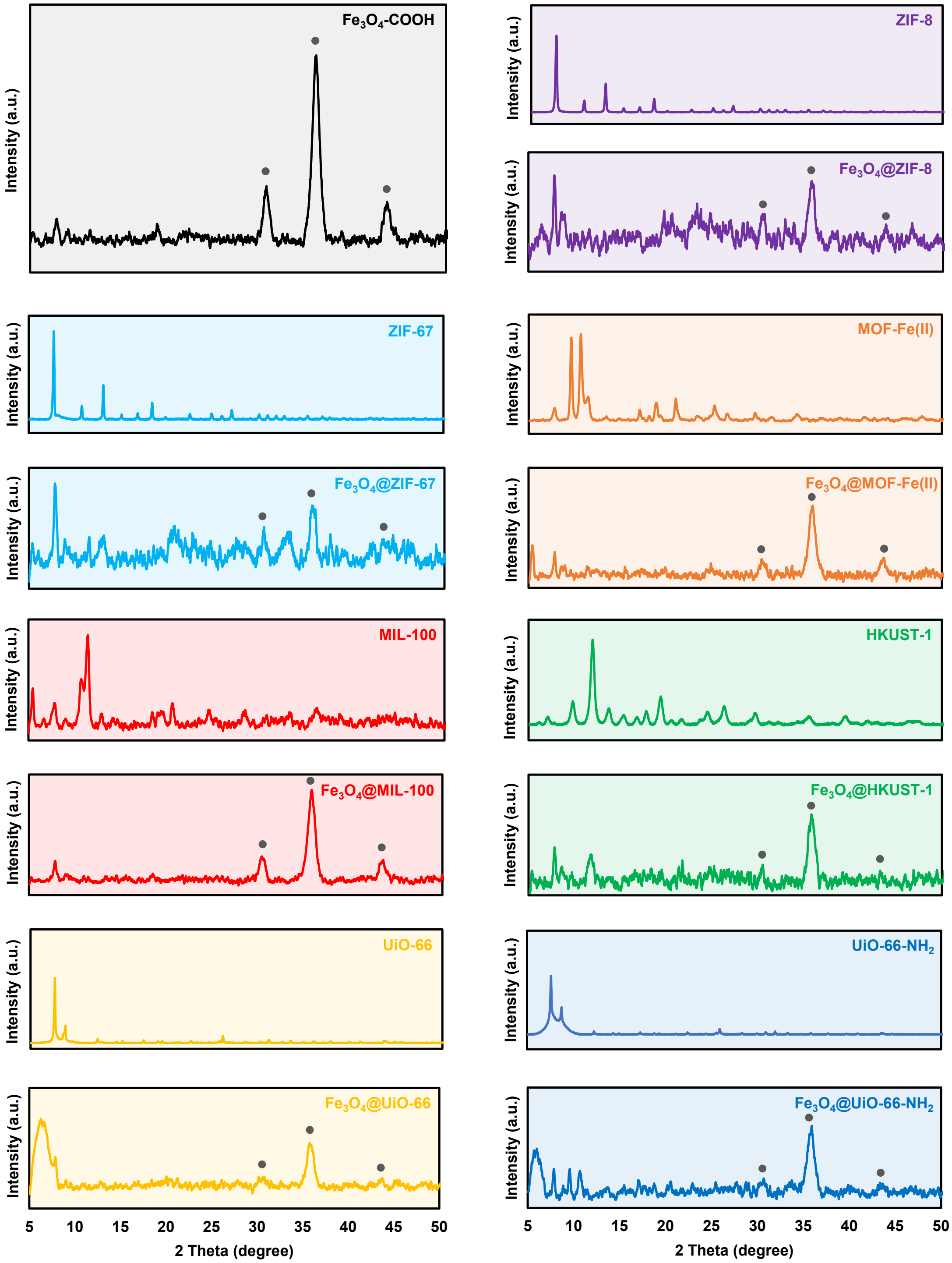

Supplementary Figure 14. XRD patterns of Fe_3O_4-COOH nanocluster, pristine MOFs, and Fe_3O_4@MOF core-shell structures. In Fe_3O_4@MOF core-shell structures, the peaks related to the acidic Fe_3O_4@COOH core are pointed out by black dots in 25-45˚ range and the rest are related to the assemble MOFs.

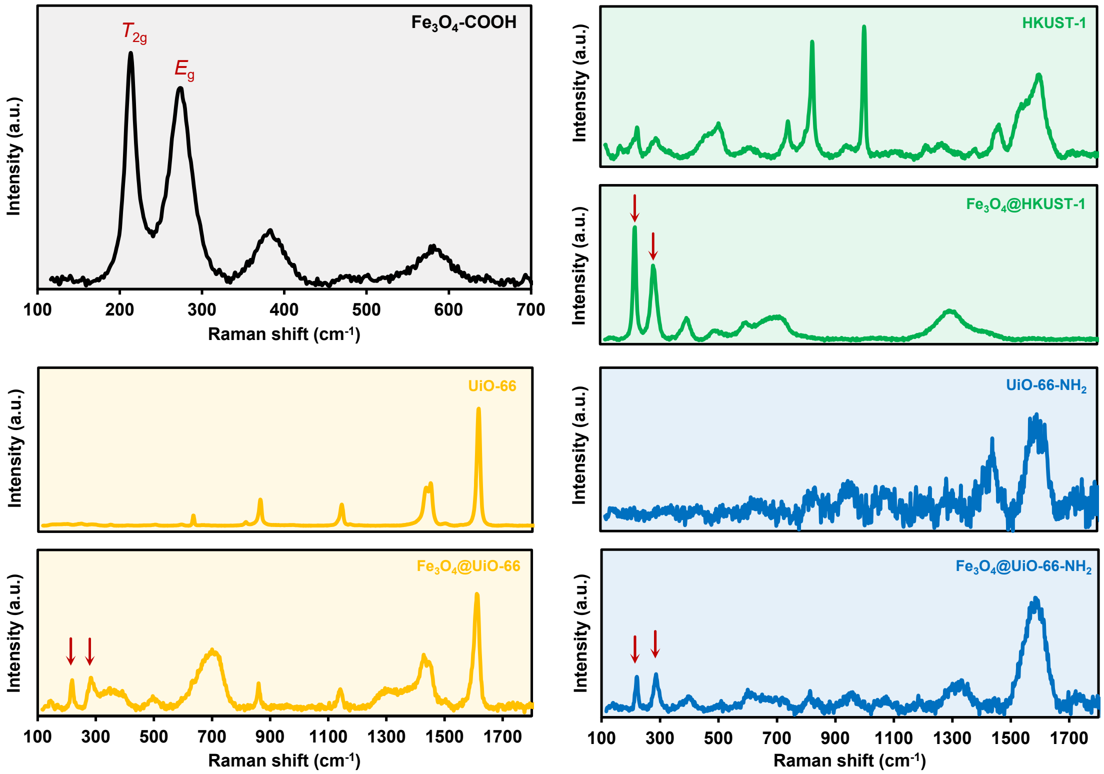

Supplementary Figure 15. Raman spectra of Fe_3O_4-COOH nanocluster, pristine MOFs, and Fe_3O_4@MOF core-shell structures. The appeared signals (pointed out by red vertical arrows, 200-300 cm^{-1} range) in Fe_3O_4@HKUST-1, Fe_3O_4@UiO-66 and Fe_3O_4@UiO-66-NH_2 are attributed to the T_{2g} (210-220 cm^{-1}) and E_g (270-280 cm^{-1}) vibrational modes of magnetic structure.

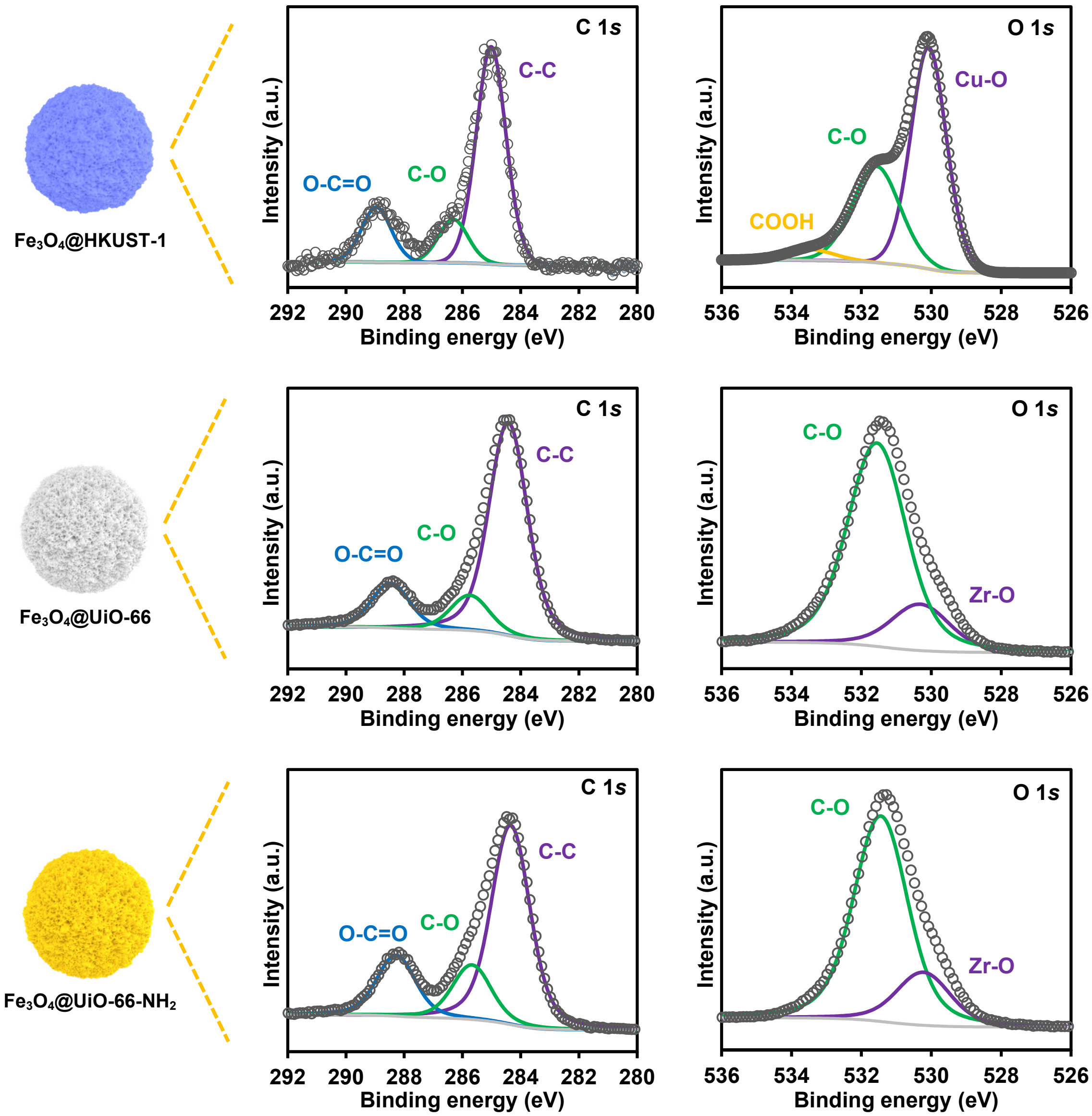

Supplementary Figure 16. High-resolution XPS spectra of water-dispersible Fe_3O_4@HKUST-1, Fe_3O_4@UiO-66 and Fe_3O_4@UiO-66-NH_2 nanomaterials. The identified C 1*s* and O 1*s* peaks of each Fe_3O_4@MOF are in accordance with their corresponding MOF spectra, showing the successful MOF coverage on the exterior surface of core-shell structure.

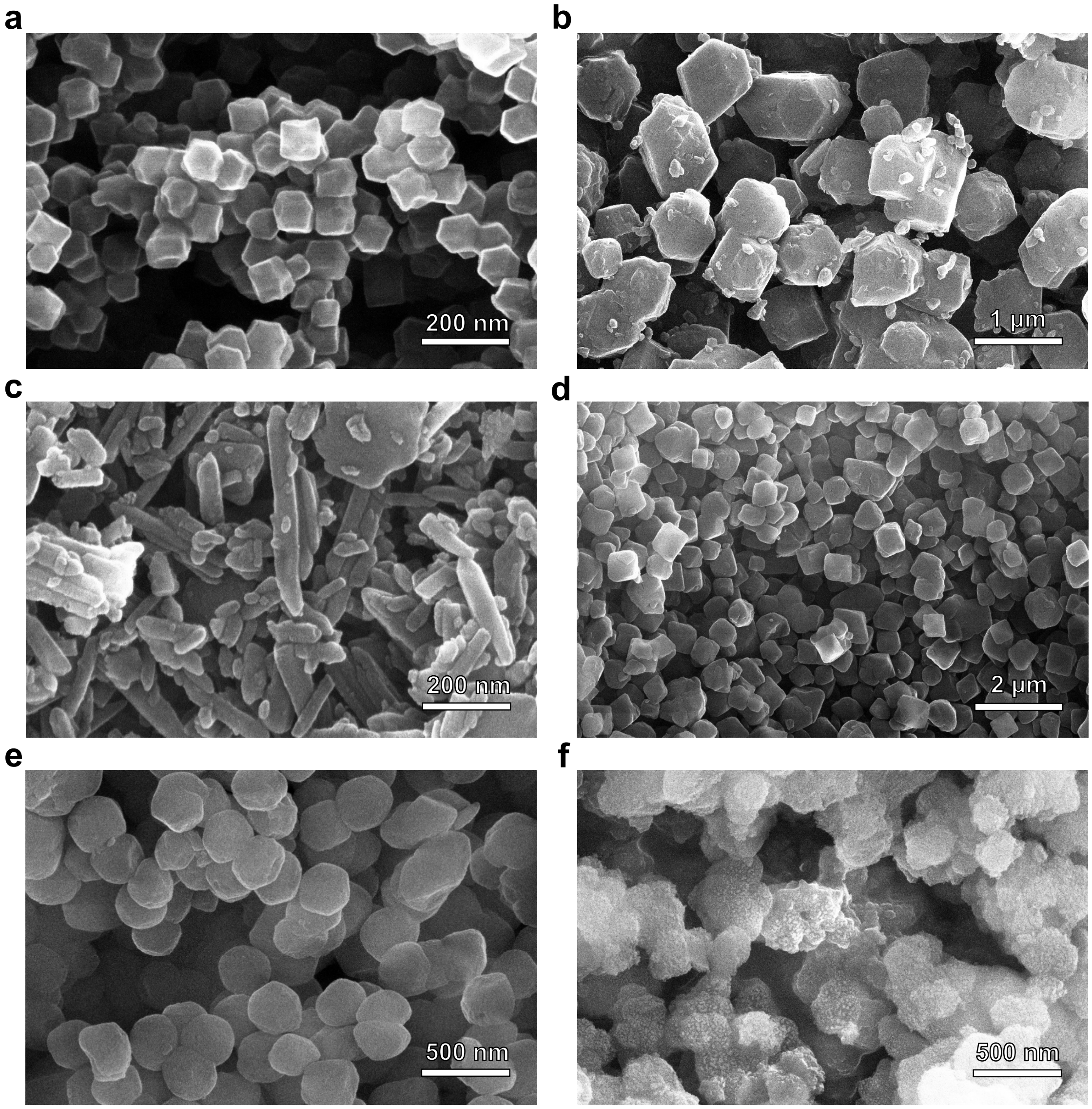

Supplementary Figure 17. SEM images of the conventional MOF crystals with different crystalline structures: (a) ZIF-8, (b) ZIF-67, (c) MOF-Fe(II), (d) MIL-100(Fe), (e) UiO-66, and (f) UiO-66-NH_2.

a b

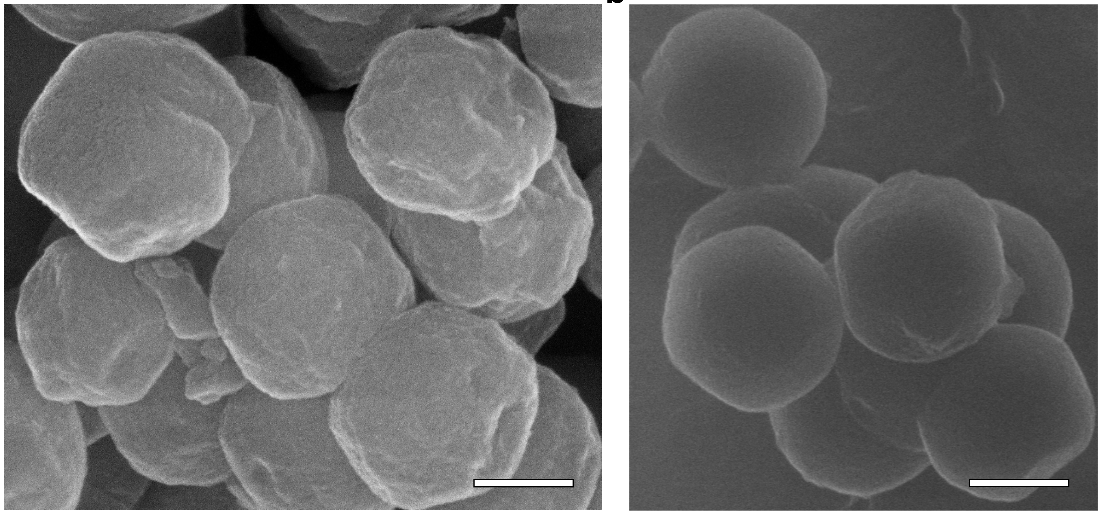

Supplementary Figure 18. High-resolution (a) SEM and (b) HIM images of zirconium-based UiO-66 metal-organic structure with an average crystal diameter of ~200-300 nm. Scale bars are 200 nm.

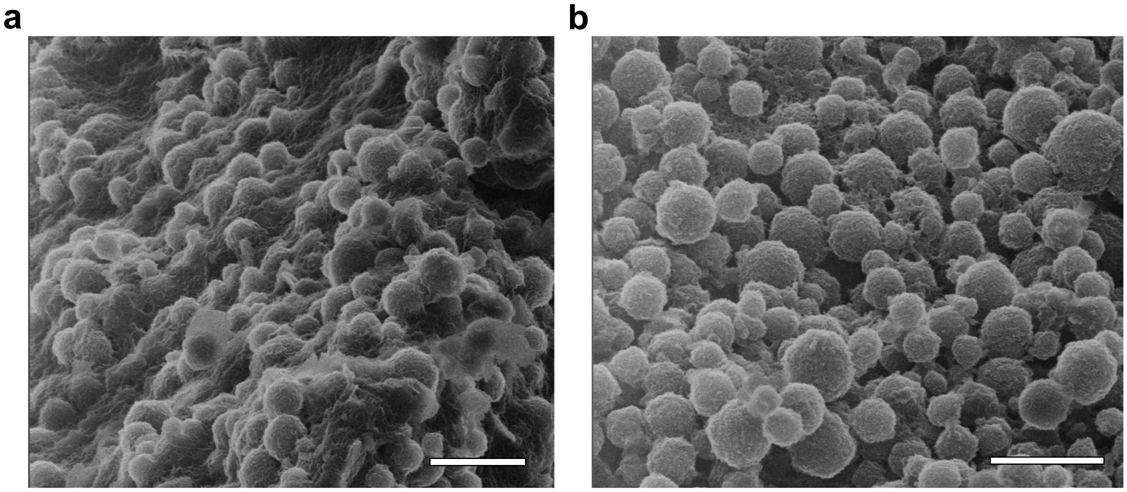

Supplementary Figure 19. Low-magnification HIM images of (a) Fe_3O_4@HKUST-1, and (b) Fe_3O_4@UiO-66 core-shell nanomaterials, depicting the variation of surface morphology as a function of the growing MOF type. Scale bars are 500 nm.

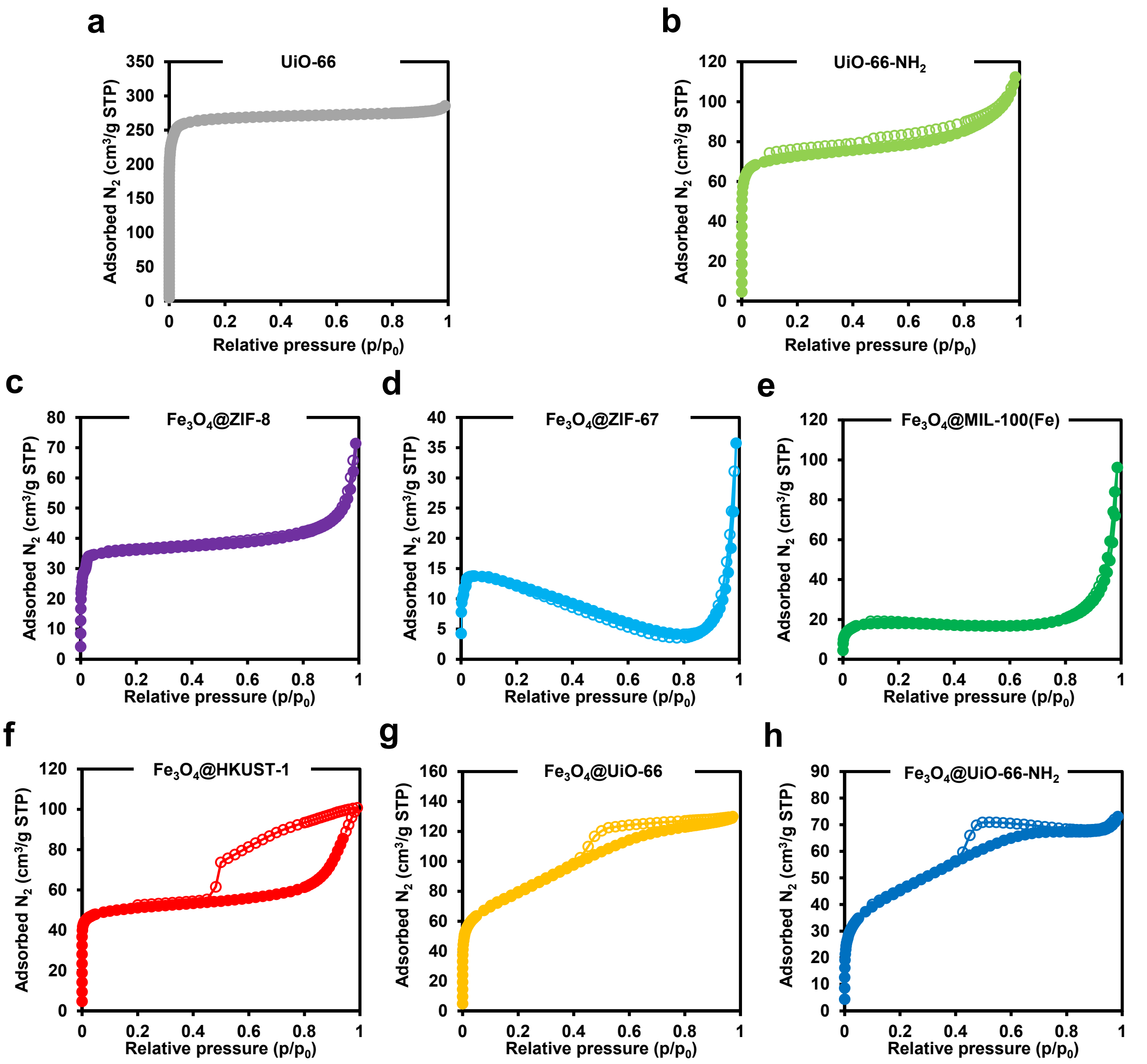

Supplementary Figure 20. Nitrogen adsorption-desorption isotherms of (a, b) Zr-based MOFs and (c-h) Fe_3O_4@MOF core-shell materials. The filled and empty circles are related to the adsorption and desorption values, respectively. The hysteresis loops and steep gas uptake at high relative pressures are a solid indicator of hierarchical micro-mesoporosity through the prepared Fe_3O_4@MOF networks.

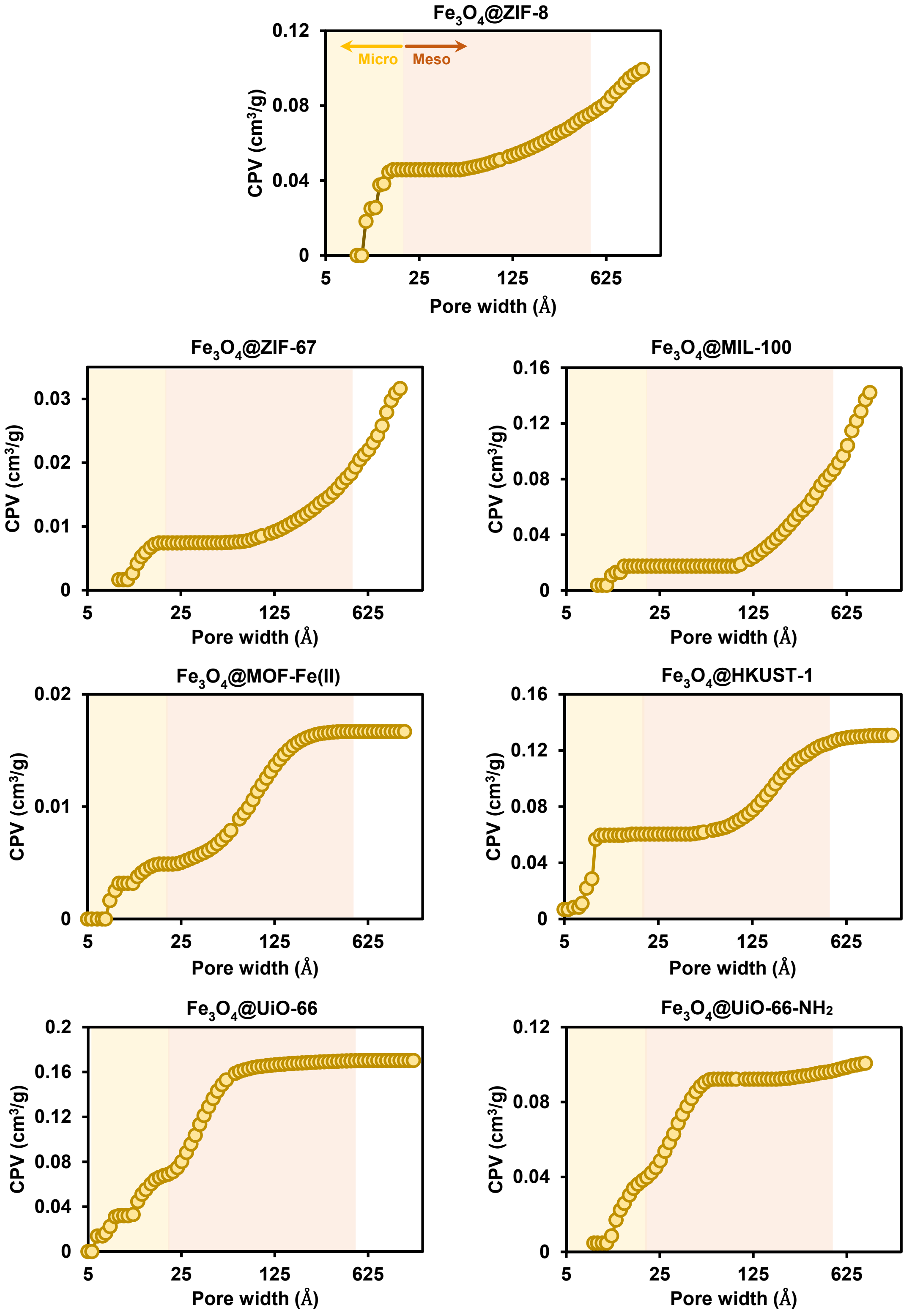

Supplementary Figure 21. Cumulative pore volume (CPV) of Fe_3O_4@MOF nanomaterials. The micropores with average pore diameter less than 2 nm and mesopores with average pore diameter between 2-50 nm have been highlighted by light gold and light brown, respectively.

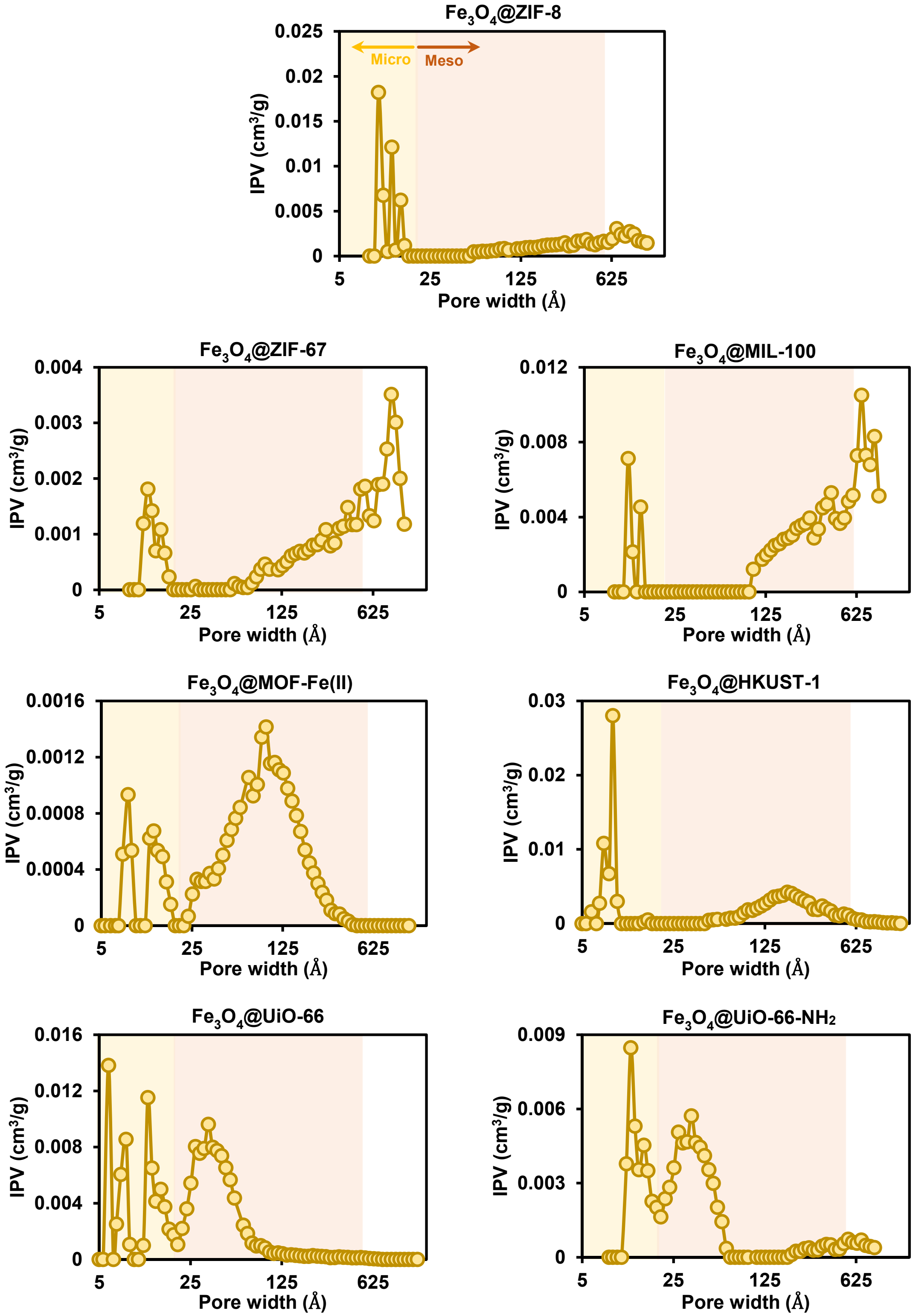

Supplementary Figure 22. Incremental pore volume (IPV) of Fe_3O_4@MOF nanomaterials. The micropores with average pore diameter less than 2 nm and mesopores with average pore diameter between 2-50 nm have been highlighted by light gold and light brown.

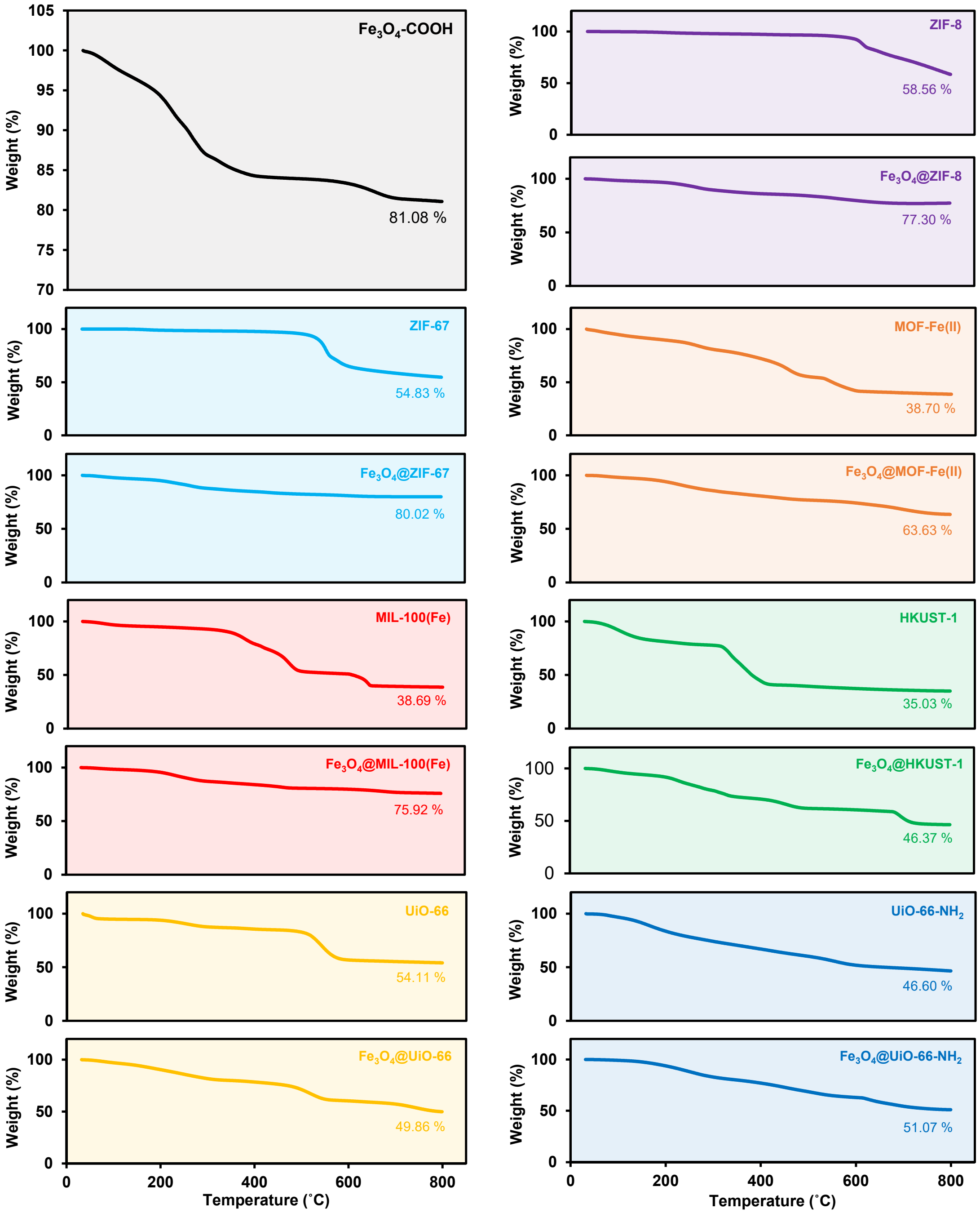

Supplementary Figure 23. TGA curves of Fe_3O_4-COOH nanocluster, pristine MOFs, and Fe_3O_4@MOF core-shell structures. All gravimetric measurements were performed under nitrogen atmosphere from room temperature to 800 ˚C.

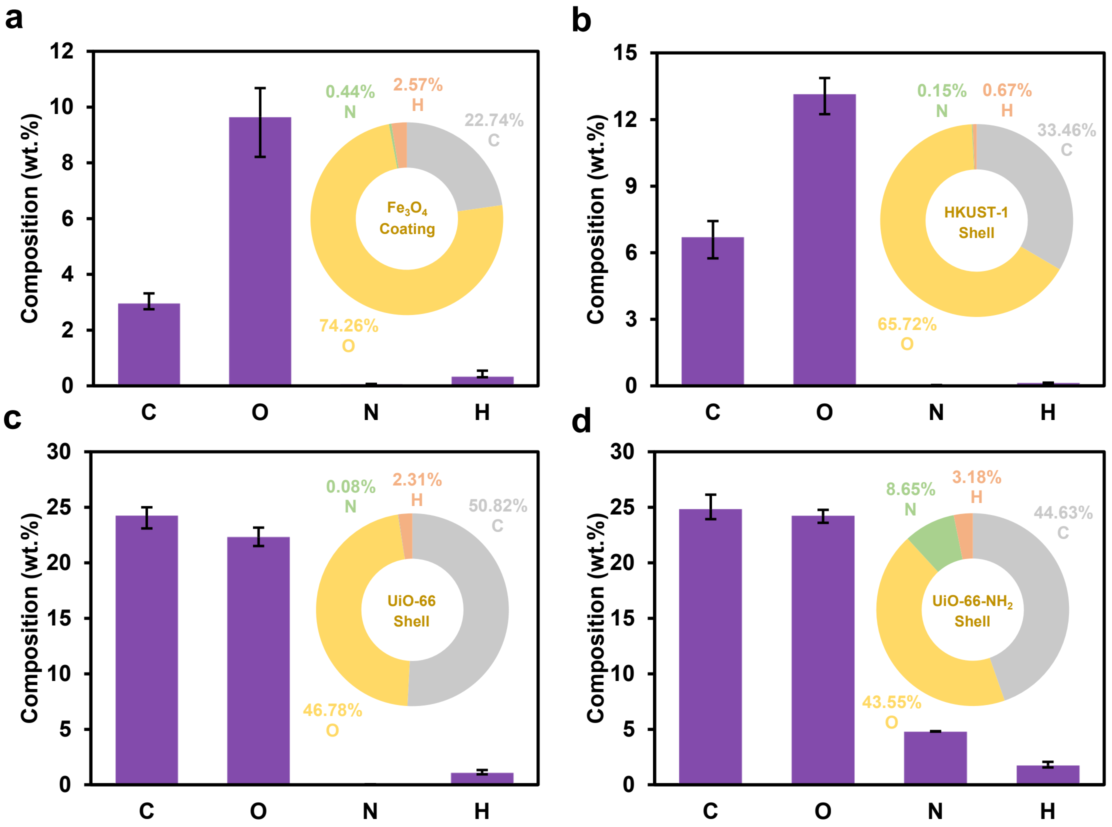

Supplementary Figure 24. Bulk elemental analysis of (a) Fe_3O_4-COOH nanoclusters, (b) Fe_3O_4@HKUST-1, (c) Fe_3O_4@UiO-66, and (d) Fe_3O_4@UiO-66-NH_2. The insets represent the elemental content of core-shell materials without considering Fe element.

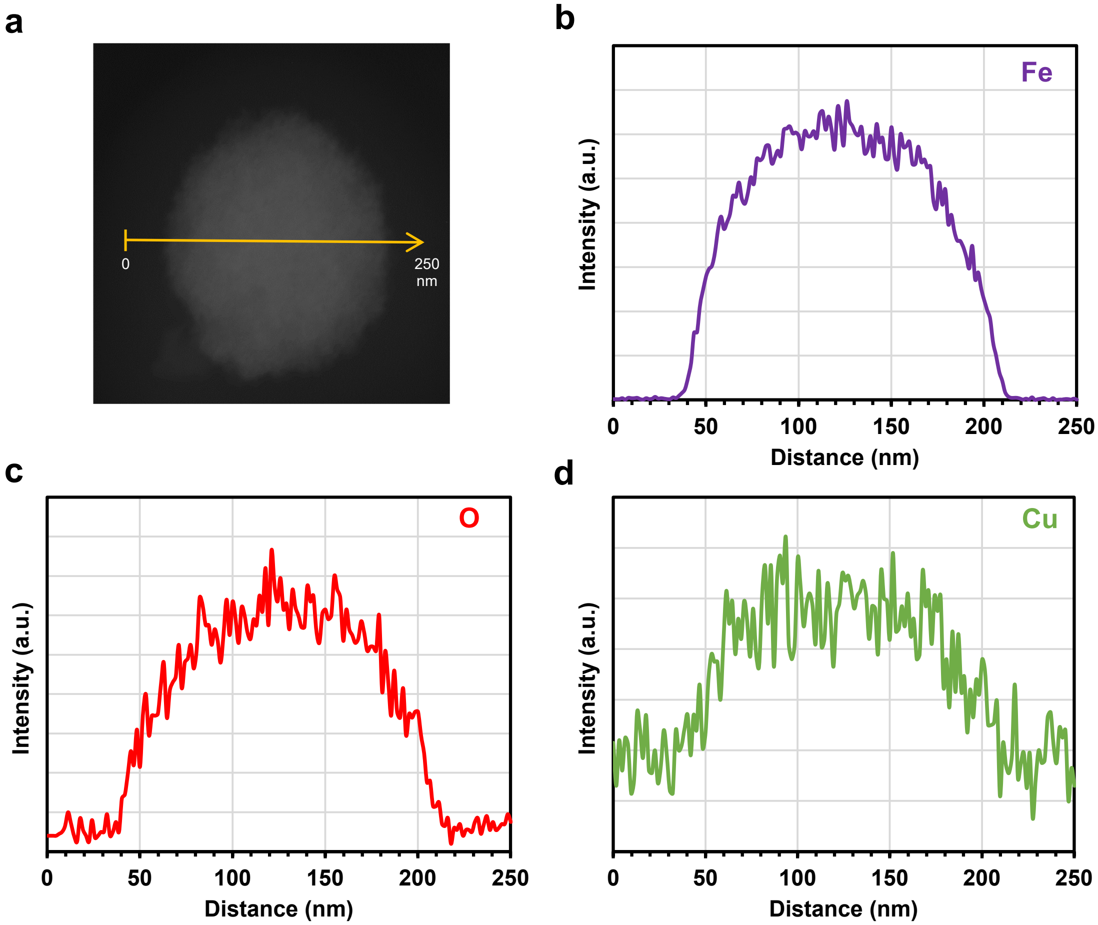

Supplementary Figure 25. (a) HAADF image and (b-d) elemental line scanning profiles (Fe, O and Cu) of Fe_3O_4@HKUST-1 core-shell material. It is observed that Cu metal ions (related to the HKUST-1 network) have evenly distrubuted on the surface magnetic nanocluster.

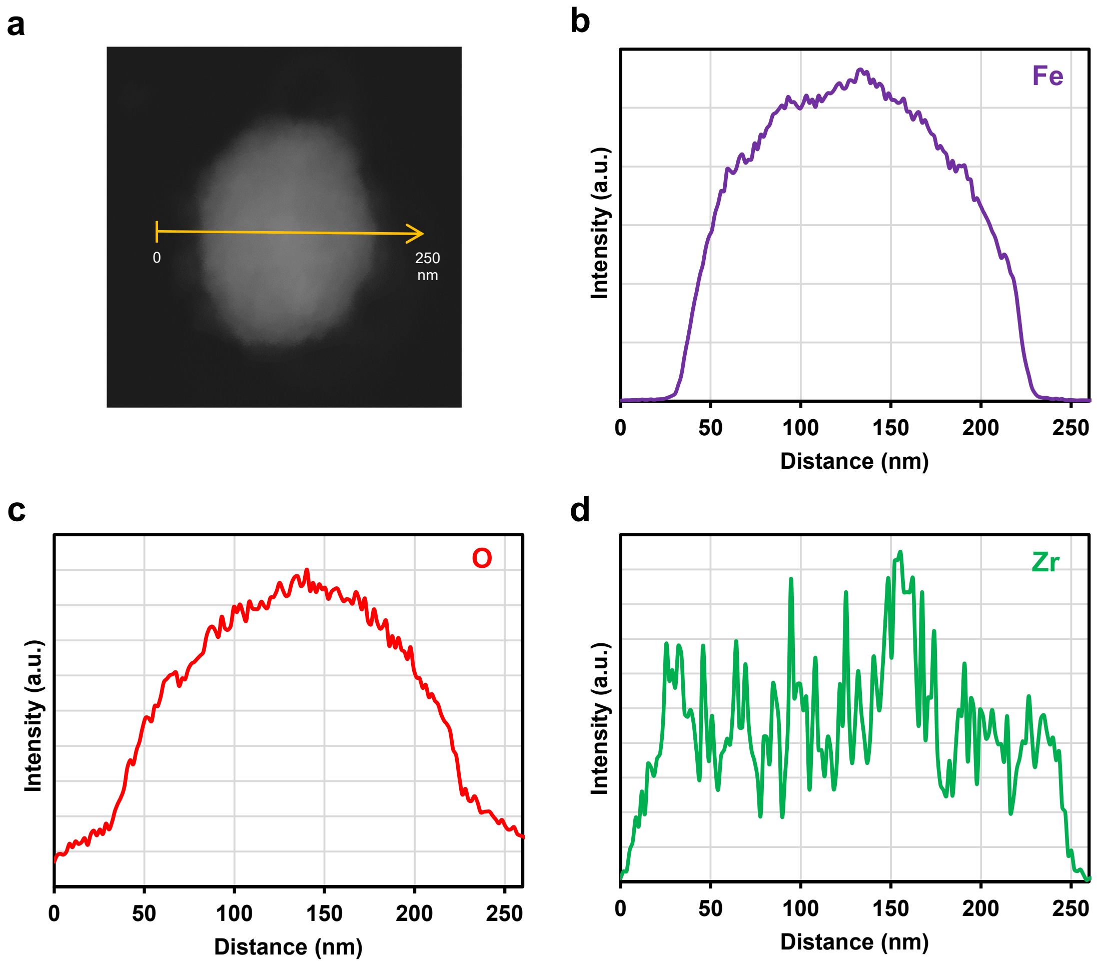

Supplementary Figure 26. (a) HAADF image and (b-d) elemental line scanning profiles (Fe, O and Zr) of Fe_3O_4@UiO-66 core-shell material. Owing to the thicker structure of UiO-66 across shell side, Zr line scanning shows amplified signals in these regions.

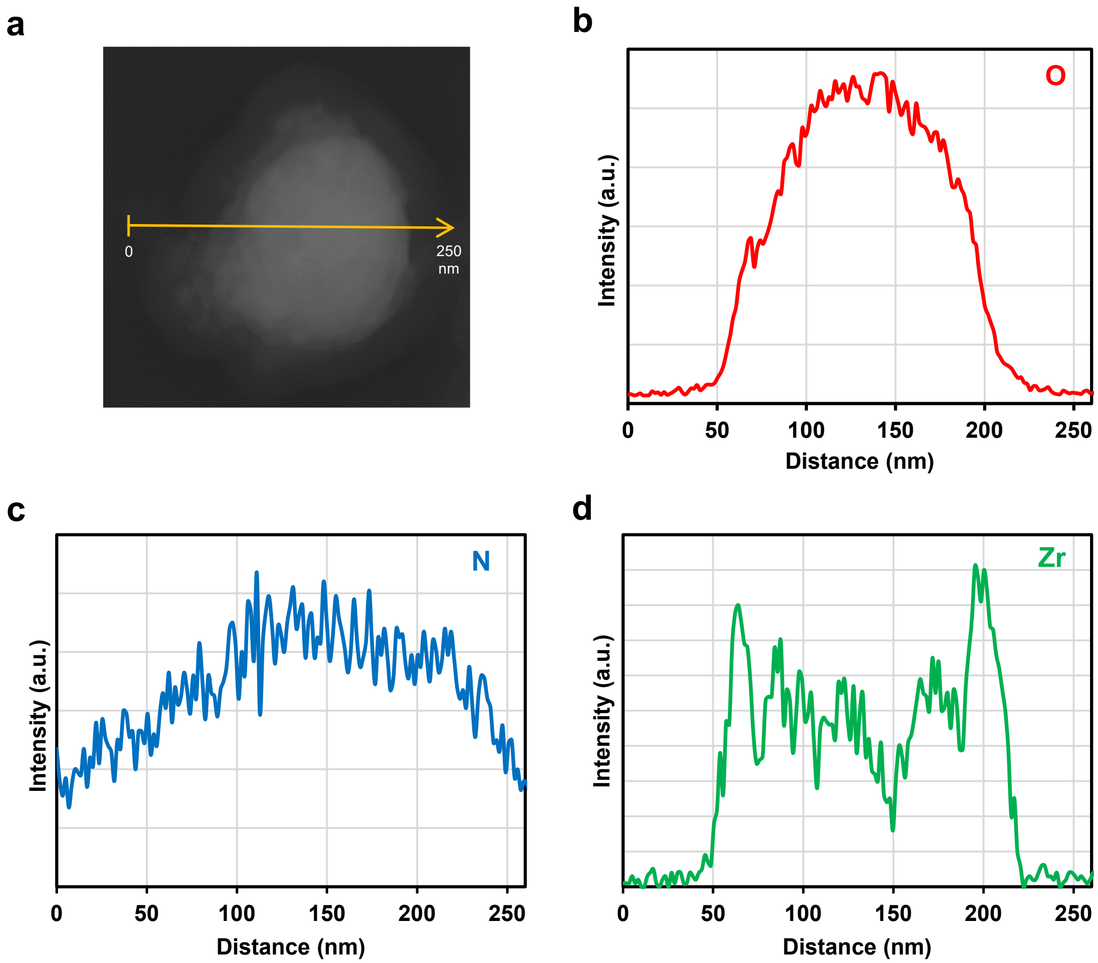

Supplementary Figure 27. (a) HAADF image and (b-d) elemental line scanning profiles (O, N and Zr) of Fe_3O_4@UiO-66-NH_2 core-shell material. The detected N elemenets are attributed to the amino groups of H_2BDC-NH_2, used in the structure UiO-66-NH_2.

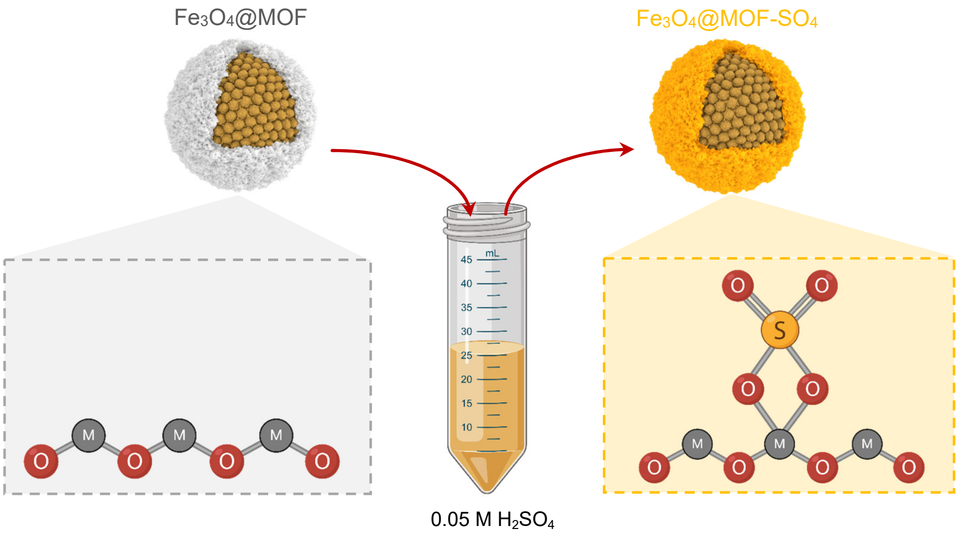

Supplementary Figure 28. Schematic representation of the sulfation process, by introducing chelating sulfate moieties through the defected structure of core-shell materials in the aqueous medium. To obtain the optimum sulfation efficiency, while avoiding the digestion of magnetic core into the acidic solvent, a 0.05 M concentration of H_2SO_4 was used. The sulfation process was performed at room temperature for 24 hr.

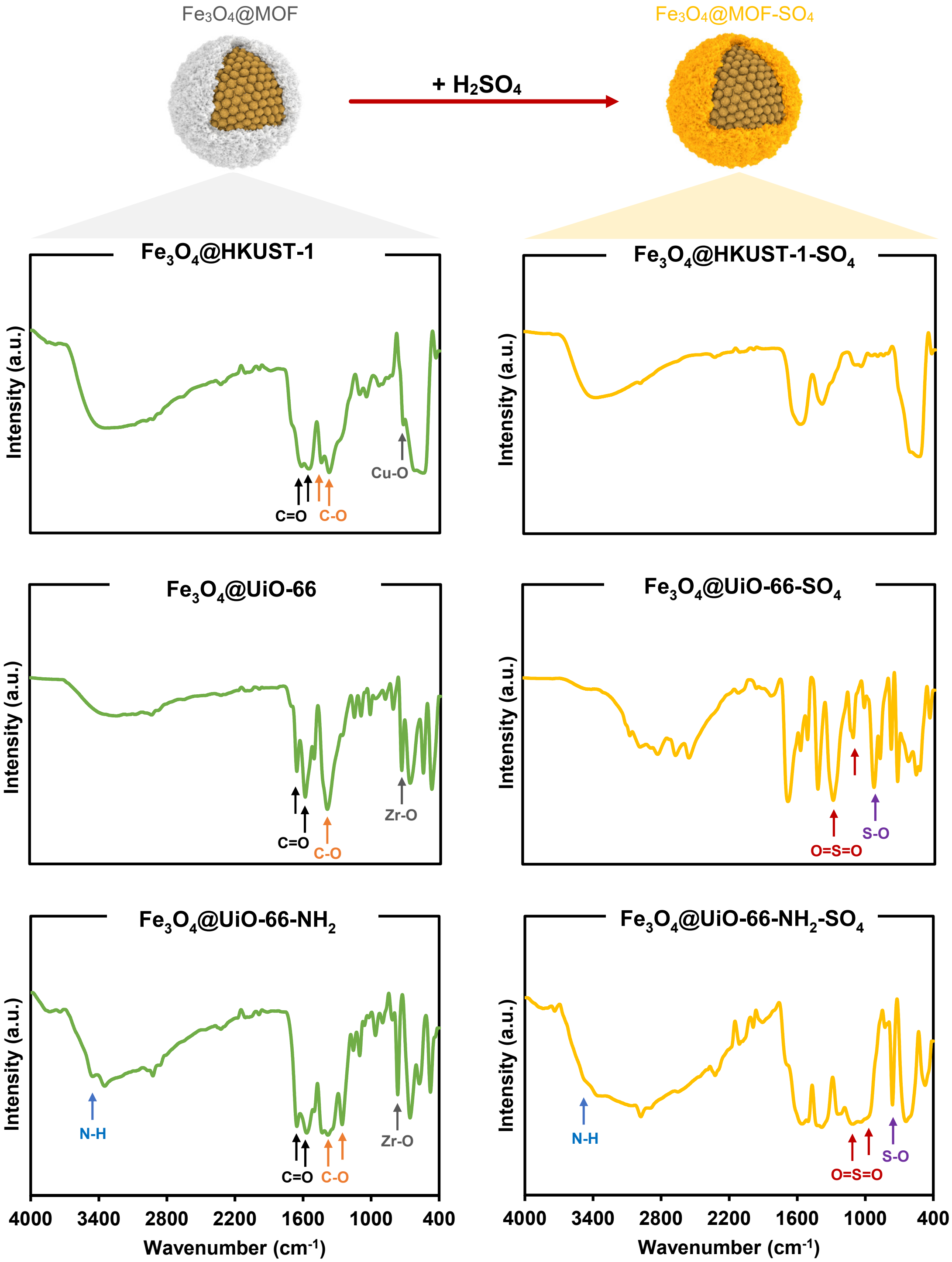

Supplementary Figure 29. FTIR analysis of Fe_3O_4@MOF and Fe_3O_4@MOF-SO_4 core-shell nanomaterials. The appeared peaks in ~800-950 cm^{-1} (S-O) and ~1000-1300 cm^{-1} (S=O) ranges confirm the successful coordination of sulfate moieties with metal centers of the water-dispersible zirconium-based core-shell materials (against HKUST-1 with negligible sulfation capability).

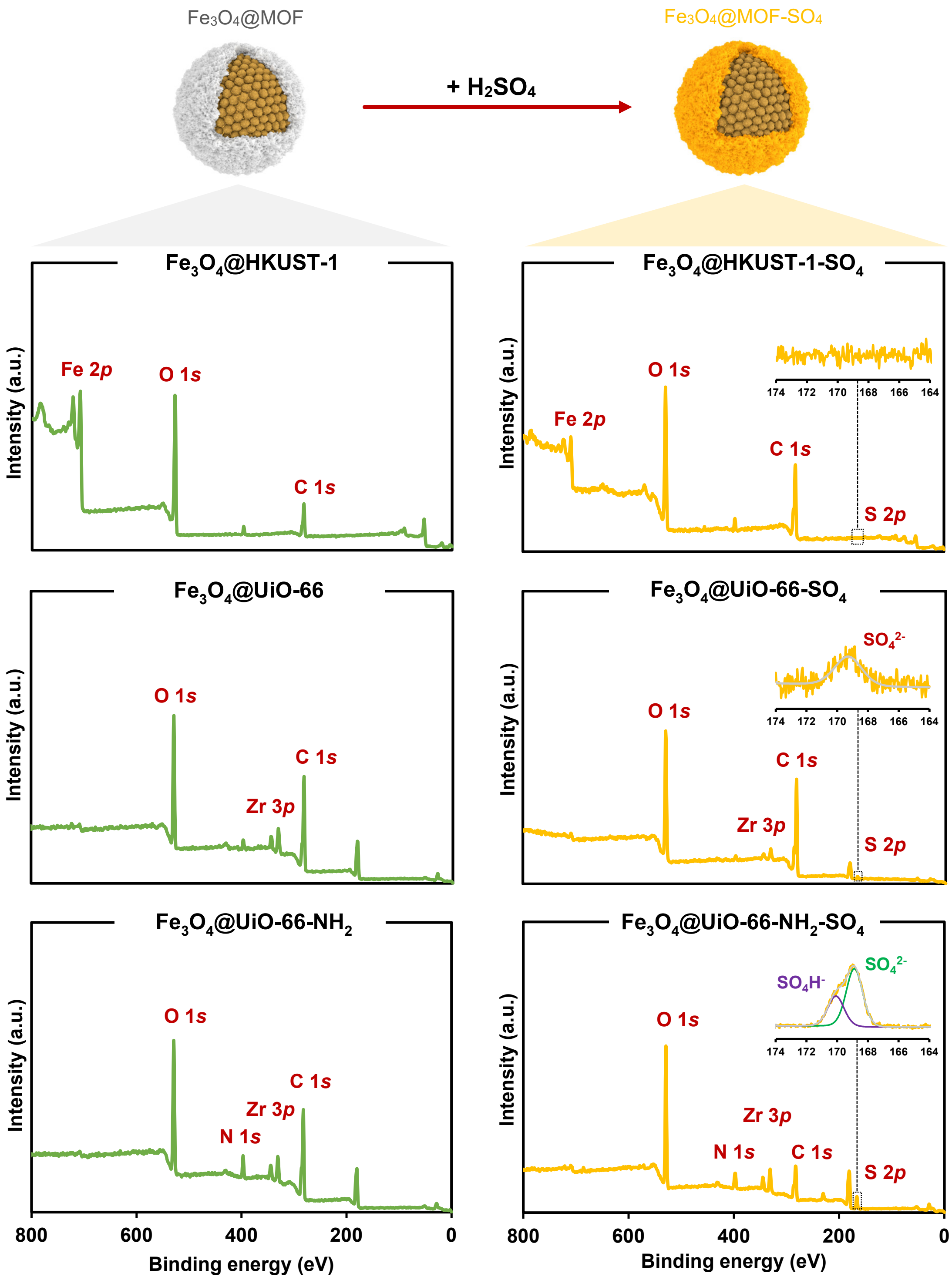

Supplementary Figure 30. XPS analysis of Fe_3O_4@MOF and Fe_3O_4@MOF-SO_4 core-shell nanomaterials. The insets indicate the absence of sulfur in Fe_3O_4@HKUST-SO_4 and the type of sulfur species (distributed on the exterior surface) in Fe_3O_4@UiO-66-SO_4 and Fe_3O_4@UiO-66-NH_2-SO_4 core-shell nanomaterials.

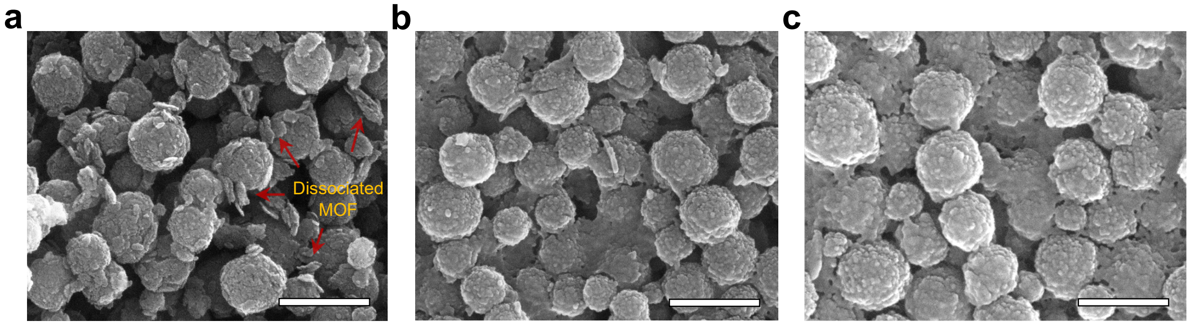

Supplementary Figure 31. SEM images of the sulfated water-dispersible nanocatalyst: (a) Fe_3O_4@HKUST-SO_4, (b) Fe_3O_4@UiO-66-SO_4, and (c) Fe_3O_4@UiO-66-NH_2-SO_4. Scale bars are 300 nm. It can be observed that Fe_3O_4@HKUST-1 structure was partially dissociated at low pH values, while Fe_3O_4@UiO-66-SO_4, and (c) Fe_3O_4@UiO-66-NH_2-SO_4 displayed a good structural stability at the same conditions.

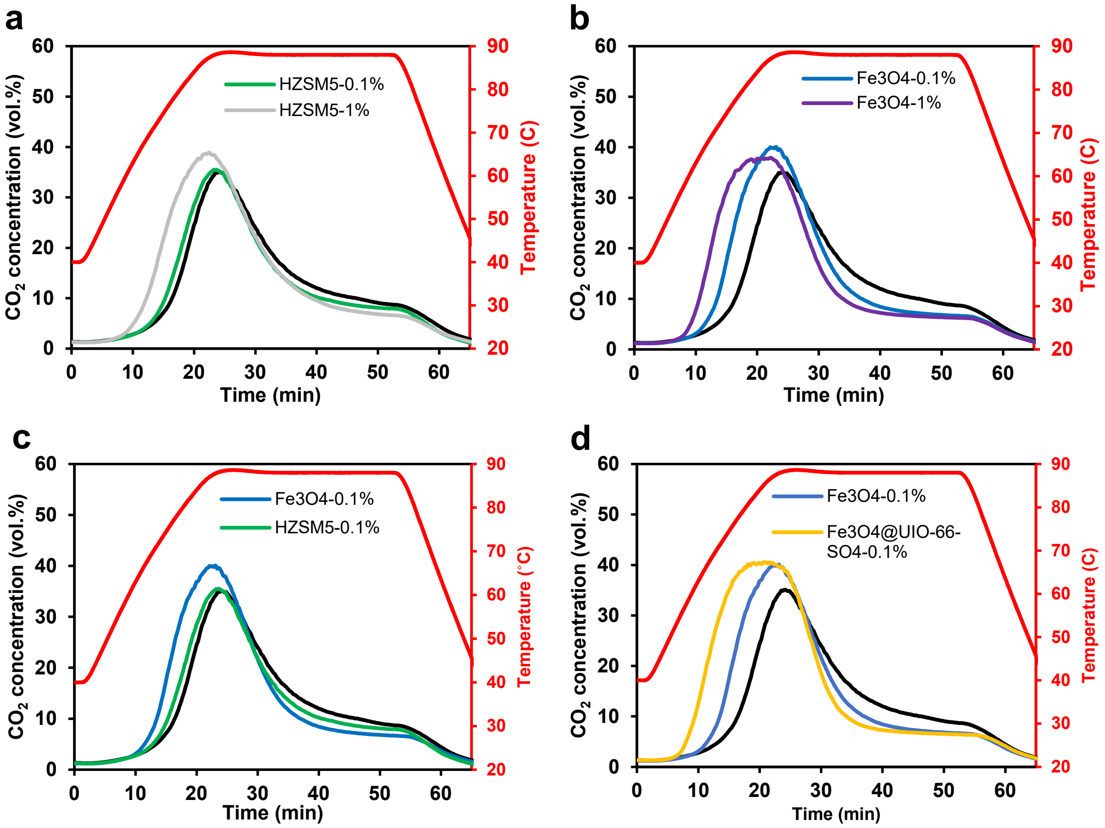

Supplementary Figure 32. CO_2 desorption profile of different acidic nanomaterials during catalytic solvent regeneration. The catalytic performance of (a) HZSM-5 (as a commercialized solid acid catalyst) and (b) Fe_3O_4-COOH (as a water-dispersible platform with acidic properties newly synthesized in this study) at different concentrations of catalyst (0.1 and 1 wt.%). Comparing the catalytic performance of (c) HZSM-5/Fe_3O_4-COOH and (d) Fe_3O_4-COOH/Fe_3O_4@UiO-66-SO_4 at low catalyst concentration of 0.1 wt.%. The solvent regeneration temperature and CO_2 desorption profile of the blank solvent (aqueous 5M MEA without using catalyst) are displayed by red and black colors, respectively, and the regeneration time was 50 min for all solvents.

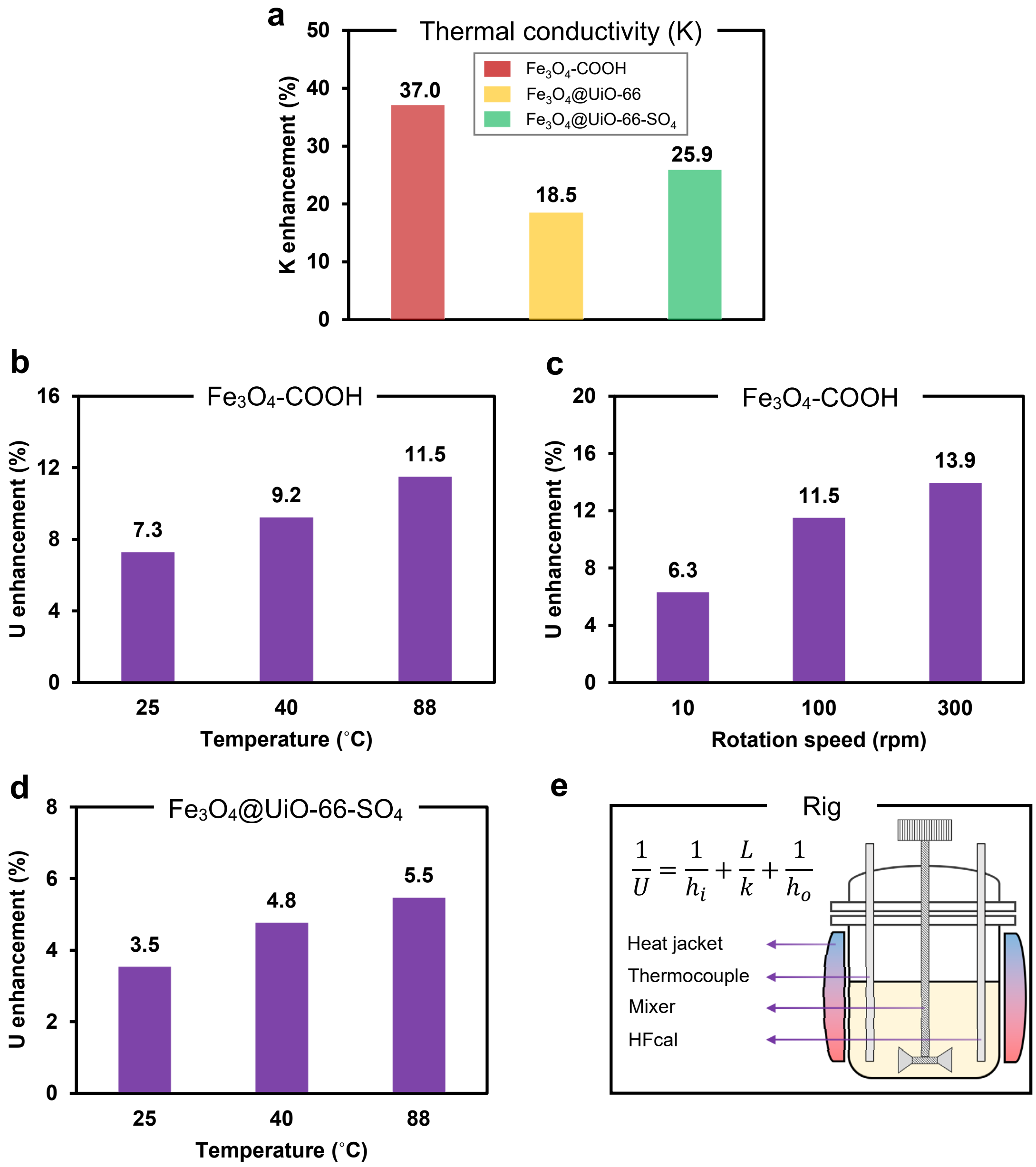

Supplementary Figure 33. Heat transfer coefficient enhancement of water in the presence of different water-dispersible nanomaterials with 0.1wt.% concentration: (a) Thermal conductivity (K) enhancement at 25 ˚C, (b) overall heat transfer (U) enhancement of Fe_3O_4-COOH at different temperatures, (c) Fe_3O_4-COOH at different rotation speeds, (d) Fe_3O_4@UiO-66-SO_4 at different temperatures, and (e) the scheme of the rig used to measure U enhancement values. The resulting K and U enhancement after adding a low concentration of Fe_3O_4-COOH and Fe_3O_4@UiO-66-SO_4 in water confirms the nanofluidic behavior of these nanomaterials.

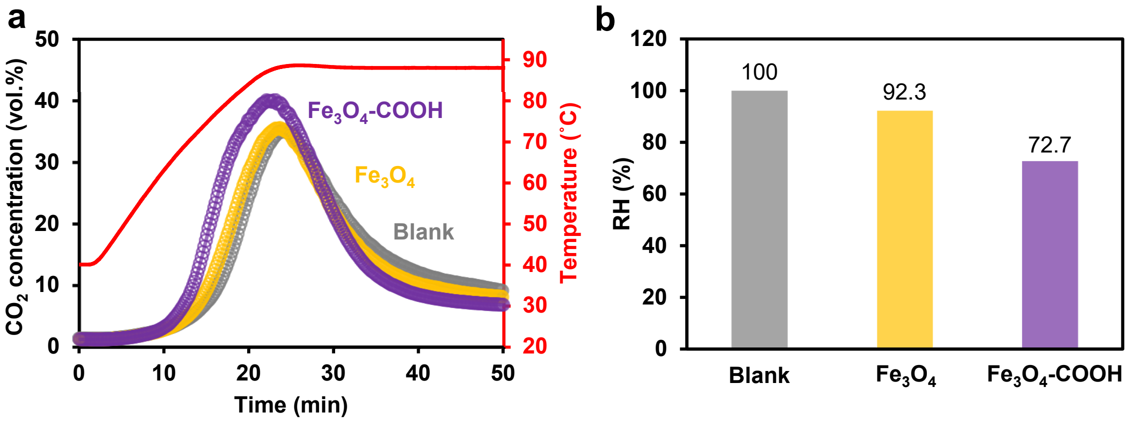

Supplementary Figure 34. (a) CO_2 desorption profile and (b) relative heat duty (RH) of 5M MEA solution in the presence of conventional Fe_3O_4 and water-dispersible Fe_3O_4-COOH nanomaterials. The concentration of nanomaterials was kept constant at 0.1 wt.% in all cases and no catalyst was used for the blank solvent. The promoted CO_2 desorption and RH reduction after adding the conventional Fe_3O_4 nanoparticles (without any chemical effects) is attributed to the positive influence of nanofluidic behavior of both heat and mass transfer coefficients.

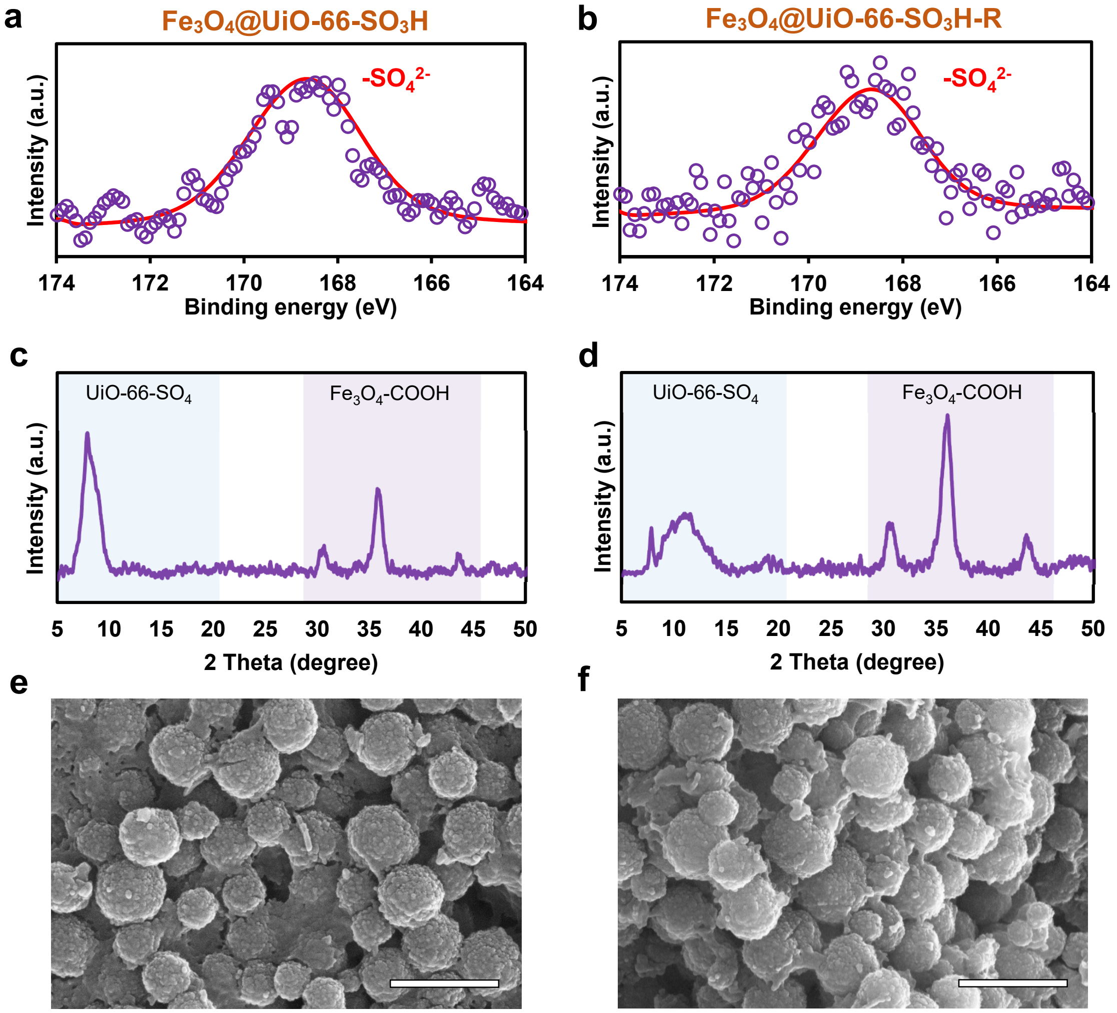

Supplementary Figure 35. Characterization of fresh Fe_3O_4@UiO-66-SO_4 and recycled Fe_3O_4@UiO-66-SO_4-R nanocatalyst: (a, b) high-resolution XPS spectra of sulfur species, (c, d) XRD patterns, and (e, f) SEM images. The scale bars of SEM images are 500 nm. It is observed that the water-dispersible Fe_3O_4@UiO-66-SO_4 nanocatalysts has acceptably maintained its physicochemical features after cyclic CO_2 absorption-desorption.

Supplementary Table 1. The results of Hammett indicator test for Fe_3O_4-COOH and Fe_3O_4@UiO-66-SO_4.

Indicators	Color		p*K*a	Hammett indicator tests	
	Acid form	Base form		Fe_3O_4-COOH	Fe_3O_4@UiO-66-SO_4
4-Phenylazoaniline	Red	Orange	+2.8	+	+
2-Nitroaniline	Red	Yellow	-0.2	+	+
4-Nitrodiphenylamine	Red	Yellow	-2.4	+	+
2,4-Dinitroaniline	Red	Yellow	-4.4	+	+
Trans-chalcone	Red	Yellow	-5.6	-	+
2-Bromo-4,6-dinitroaniline	Red	Yellow	-6.6	-	+
Anthraquinone	Yellow	Colorless	-8.1	-	+
4-Nitrotoluene	Yellow	Colorless	-11.4	-	+
4-Nitrofluorobenzene	Yellow	Colorless	-12.4	-	+
2,4-Dinitrotoluene	Yellow	Colorless	-13.8	-	+
2,4-Dinitrofluorobenzene	Yellow	Colorless	-14.5	-	+

Supplementary References

1. Dheyab, M. A. et al. Simple rapid stabilization method through citric acid modification for magnetite nanoparticles. *Sci. Rep.* **10**, 10793 (2020).
2. Zhang, X. et al. SO_4^{2-}/ZrO_2 supported on γ-Al_2O_3 as a catalyst for CO_2 desorption from CO_2-loaded monoethanolamine solutions. *AIChE J.* **64**, 3988-4001 (2018).
3. Gao, H. et al. Catalytic performance and mechanism of SO_4^{2-}/ZrO_2/SBA-15 catalyst for CO_2 desorption in CO_2-loaded monoethanolamine solution. *Appl. Energy* **259**, 114179 (2020).
4. García-Palacín, M. et al. Sized-controlled ZIF-8 nanoparticle synthesis from recycled mother liquors: environmental impact assessment. *ACS Sustain. Chem. Eng.* **8**, 2973-2980 (2020).
5. Meng, H. et al. ZIF67@MFC-derived Co/N-C@CNFs interconnected frameworks with graphitic carbon-encapsulated Co nanoparticles as highly stable and efficient electrocatalysts for oxygen reduction reactions. *ACS Appl. Mater. Interfaces* **12**, 41580-41589 (2020).
6. Nivetha, R. et al. Highly porous MIL-100(Fe) for the hydrogen evolution reaction (HER) in acidic and basic media. *ACS Omega* **5**, 18941-18949 (2020).
7. Reyhani, A., Mazaheri, O., Alivand, M. S., Mumford, K. A. & Qiao, G. Temporal control of RAFT polymerization via magnetic catalysis. *Polym. Chem.* **11**, 2838-2846 (2020).
8. Kwon, O. et al. Computer-aided discovery of connected metal-organic frameworks. *Nat. Commun.* **10**, 3620 (2019).
9. Abánades Lázaro, I., Wells, C. J. R. & Forgan, R. S. Multivariate modulation of the Zr MOF UiO-66 for defect-controlled combination anticancer drug delivery. *Angew. Chem. Int. Ed.* **59**, 5211-5217 (2020).
10. Schaate, A. et al. Modulated synthesis of Zr-based metal–organic frameworks: from nano to single crystals. *Chem. Eur. J.* **17**, 6643-6651 (2011).
11. Alivand, M. S. et al. Data in brief on CO_2 absorption-desorption of aqueous-based amino acid solvents with phase change behaviour. *Data in Brief* **27**, 104741 (2019).
12. Alivand, M. S. et al. Development of aqueous-based phase change amino acid solvents for energy-efficient CO_2 capture: The role of antisolvent. *Appl. Energy* **256**, 113911 (2019).
